# *NAL8* encodes a prohibitin that contributes to leaf and spikelet development by regulating mitochondria and chloroplasts stability in rice

**DOI:** 10.1186/s12870-019-2007-4

**Published:** 2019-09-11

**Authors:** Ke Chen, Tao Guo, Xin-Min Li, Yi-Bing Yang, Nai-Qian Dong, Chuan-Lin Shi, Wang-Wei Ye, Jun-Xiang Shan, Hong-Xuan Lin

**Affiliations:** 1 National Key Laboratory of Plant Molecular Genetics, CAS Centre for Excellence in Molecular Plant Sciences and Collaborative Innovation Center of Genetics & Development, Shanghai Institute of Plant Physiology & Ecology, Shanghai Institute for Biological Sciences, Chinese Academic of Sciences, Shanghai, 200032 China; 2 University of the Chinese Academy of Sciences, Beijing, 100049 China; 3 School of Life Science and Technology, ShanghaiTech University, Shanghai, 201210 China

**Keywords:** Prohibitin subunit, Leaf morphology, Grain number, Proteomics, Rice

## Abstract

**Background:**

Leaf morphology and spikelet number are two important traits associated with grain yield. To understand how genes coordinating with sink and sources of cereal crops is important for grain yield improvement guidance. Although many researches focus on leaf morphology or grain number in rice, the regulating molecular mechanisms are still unclear.

**Results:**

In this study, we identified a prohibitin complex 2α subunit, NAL8, that contributes to multiple developmental process and is required for normal leaf width and spikelet number at the reproductive stage in rice. These results were consistent with the ubiquitous expression pattern of *NAL8* gene. We used genetic complementation, CRISPR/Cas9 gene editing system, RNAi gene silenced system and overexpressing system to generate transgenic plants for confirming the fuctions of *NAL8*. Mutation of *NAL8* causes a reduction in the number of plastoglobules and shrunken thylakoids in chloroplasts, resulting in reduced cell division. In addition, the auxin levels in *nal8* mutants are higher than in TQ, while the cytokinin levels are lower than in TQ. Moreover, RNA-sequencing and proteomics analysis shows that *NAL8* is involved in multiple hormone signaling pathways as well as photosynthesis in chloroplasts and respiration in mitochondria.

**Conclusions:**

Our findings provide new insights into the way that *NAL8* functions as a molecular chaperone in regulating plant leaf morphology and spikelet number through its effects on mitochondria and chloroplasts associated with cell division.

**Electronic supplementary material:**

The online version of this article (10.1186/s12870-019-2007-4) contains supplementary material, which is available to authorized users.

## Background

Rice is the world’s most important cereal crop because it feeds more than 50% of human population every day [1]. Traditionally, tiller number, grain weight, and the number of grains per panicle are considered to be the main factors that determine grain yield [2]. The size and shape of the plant leaf is also an important agronomic trait associated with photosynthetic efficiency [3]. To uncover the molecular mechanisms that determine the balance between leaf size and shape of the source and spikelet numbers in the sink in rice, numerous studies have focused on identifying quantitative trait loci (QTLs) that contribute to these processes. Many genes and pathways have been identified recently, confirming that multiple plant hormone signaling pathways, miRNAs and transcription factors are involved in maintenance of reproductive meristem activity [4].

Leaf size and leaf rolling are key components in plant architecture associated with crop yield [5]. Abiotic stresses such as temperature, salt, UV radiation and toxic heavy metals can affect leaf morphology and further interfere with the light reception, carbon fixation, carbon assimilation, and photosynthetic rate [6]. Recently, several studies concerning leaf size and shape have been reported in rice [3, 7–10], highlighting the association between leaf morphology for photosynthesis and rice grain production. In particular, several QTLs associated with grain number and plant development have been identified and characterized in rice [11–22]; these genes are involved in cell differentiation and cell proliferation through various phytohormone- mediated signaling transduction.

Prohibitins (PHBs) are ubiquitous and highly conserved proteins in eukaryotes [23], that exist as complexes comprised of two highly homologous subunits, PHB1 and PHB2. In previously studies, PHB1 was identified as a potential tumor suppressor that contributes to anti-proliferative activity, and PHB2 was identified as a repressor of nuclear estrogen receptor activity, which led to PHBs being identified as potential targets for drug discovery and medical applications [24]. Between 12 and 16 PHBs bind to each other to form a ring-like heterodimer structure in the mitochondrial inner membrane, which serves to stabilize the mitochondrial genome [25]. In addition, an earlier report suggested that prohibitin is indispensable for the activation of the Raf–MEK–ERK pathway by Ras [26]. In *Arabidopsis*, there are seven conserved *PHB* genes [27] that are associated with meristem development. The *Arabidopsis PHB3* gene displays multiple functions in ethylene-induced gene expression [28], nitric oxide (NO) accumulation and response [29], salicylic acid (SA) biosynthesis induction [30], quiescent center cell (QC) and distal stem cell (DSC) identity [31], and root meristem cell proliferation [32, 33]. Furthermore, *OsPHB1* was shown to be involved in cell death and senescence through the formation dimers in the defense reaction and programmed cell death (PCD) in rice [34].

To investigate the molecular mechanisms underlying the regulation of leaf width and grain numbers, we performed an ethyl methanesulfonate (EMS) mutation screen in the *indica* rice variety ‘TeQing’ (TQ), and isolated a narrow leaf-width and reduced grain-number mutant, which we named *narrow leaf 8* (*nal8*). We characterized *NAL8*, a gene that encodes a prohibitin 2α subunit that functions as a molecular chaperone in rice. Transgenic assays confirmed that *NAL8* is responsible for controlling leaf width and grain number in rice. Moreover, chloroplastic ultrastructure and the subcellular structure of leaf vascular bundles were severely altered in the *nal8* mutant, suggesting that *NAL8* is involved in the regulation of photosynthetic efficiency and cell division. RNA-sequencing (RNA-seq) and proteomics analysis demonstrated that NAL8 is an important regulator of photosynthesis and respiration, and that it possesses the potential to improve yield in rice breeding programs.

## Results

### Characterization of the nal8 mutant and identification of NAL8

**Fig. 1:**
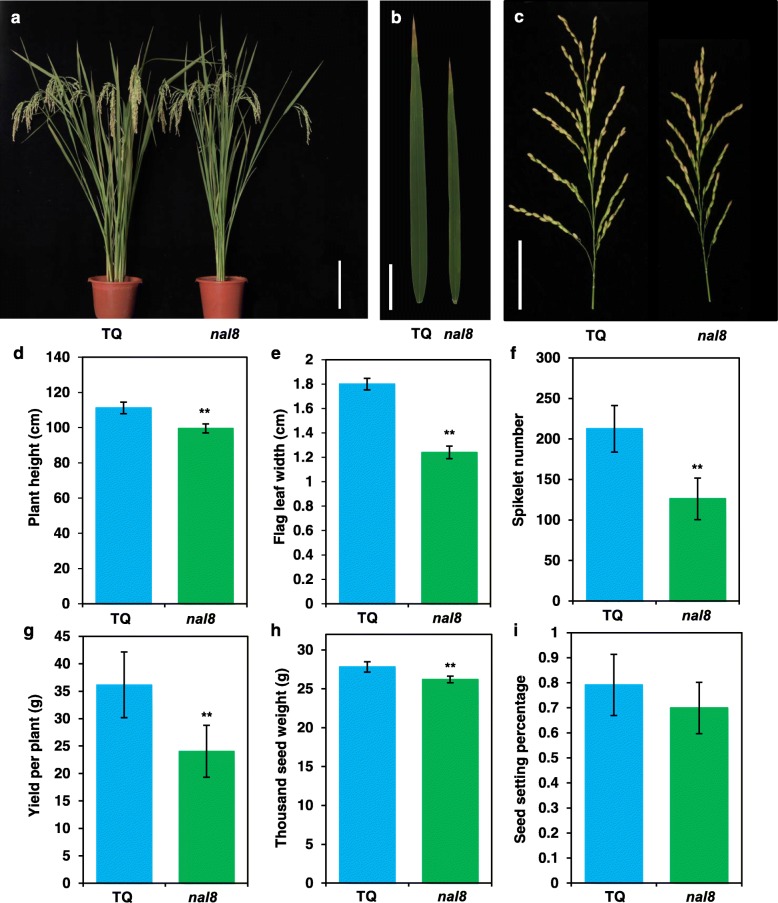
Morphology of *nal8* mutant plants showing the narrow leaf width and reduced spikelet number. **a** Plant architecture of the wild-type TQ and *nal8* mutant at the reproductive stage. Scale bar = 15 cm. **b** Flag leaves of TQ and *nal8* mutants. Scale bar = 5 cm. **c** Mature panicles of TQ and the *nal8* mutant. Scale bar = 10 cm. **d**-**i** Comparisons of **d** the average plant height, **e** average flag leaf width, **f** average spikelet number, **g** average yield per plant, **h** average thousand seed weight, and **i** average seed setting percentage between TQ and the *nal8* mutant. *n* = 20 plants for all six comparisons. Values in **d**-**i** are given as the mean ± SD. *P < 0.05; **P < 0.01 compared with the corresponding TQ wild-type using Student’s *t*-test

The *nal8* mutant was isolated from an EMS mutant library of the elite *Oryza sativa indica* variety TQ. The mutant displayed reduced leaf width and spikelet number, and slightly reduced plant height (Fig. 1a, b, c). Considering that leaf width is the most significant phenotype of the mutant, we named it *nal8*. We also measured many plant traits and found that the average plant height, average flag leaf width, and average spikelet number are significantly reduced in *nal8* mutant plants compared to TQ (Fig. 1d, e, f). In addition, the average yield and average thousand seed weight are also significantly different between TQ and the *nal8* mutant (Fig. 1g, h). Although grain yield in the *nal8* mutant is obviously reduced, the seed setting rates of TQ and *nal8* mutant plants are similar, indicating that *NAL8* is not involved in the fertilization process in rice (Fig. 1i). We then compared the grain size and flag leaf length, and found that there were no differences in average grain width and average flag leaf length compared to TQ (Additional file 1: Figure S1A-E). The phenotypes of *nal8* plants suggest that the *NAL8* gene plays an essential role in morphogenesis in rice.

**Fig. 2:**
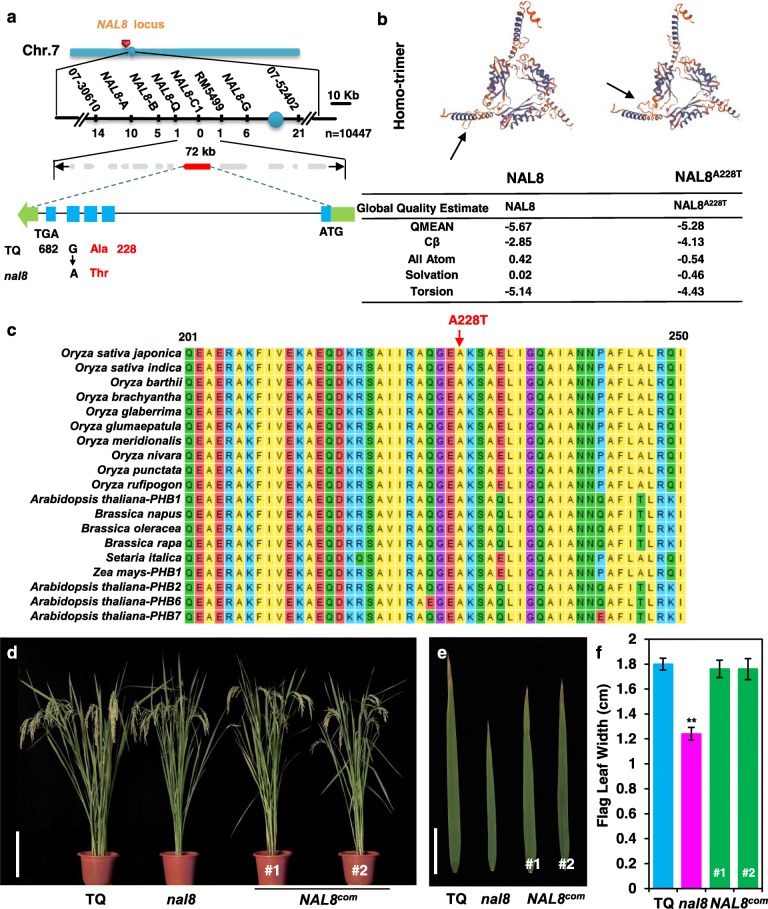
Map-based cloning of *NAL8* and genetic complementation. **a** The *NAL8* locus was initially mapped to chromosome 7 between 07 and 30,610 and 07–52,402 marker loci, and was further delimited to a 72-kb region between marker loci NAL8-Q and RM5499 that contains 12 open reading frames. Numbers below the marker loci indicate the number of recombinants. The *NAL8* gene is predicted to have five exons (blue boxes), and the green arrows indicate the direction of transcription on chromosome 7. The start (ATG) and stop (TGA) codons are indicated as well. A single nucleotide mutation (a G to A transition) in *NAL8* causes a predicted alanine to threonine change at amino acid position 228. **b** The predicted protein structures of NAL8 and NAL8^A228T^ determined by SWISS-MODEL. Arrows indicate the potential structural site changes in the homo-trimers. The table shows the protein quality estimates for the two predicted structures. **c** Amino acid sequence alignment of the partial SPFH domain in predicted prohibitin protein sequences from monocot and dicot species. The red arrow indicates the mutated site between TQ and the *nal8* mutant, which is highly conserved in all examined plant prohibitins. **d** Plant architecture at the reproductive stage of the *NAL8* transgenic complementation lines *NAL8*^*com*^#1 and #2 in the *nal8* mutant background compared with TQ and the *nal8* mutant Scale bar = 15 cm. **e** Leaves of TQ, *nal8* and the transgenic complementation lines *NAL*^*com*^#1 and #2. Scale bar = 5 cm. **f** Statistical analysis of average flag leaf lengths in TQ, *nal8* and the transgenic complementation lines *NAL*^*com*^#1 and #2. Values are given as the mean ± SD. *P < 0.05; **P < 0.01 compared with the corresponding TQ control using Student’s *t*-test in **f**

To identify the candidate *NAL8* gene, we generated an F_2_ population derived from a cross between the *nal8* mutant line and the *japonica* rice variety ‘Jiahua-1’. The ratio of normal flag leaf width to narrow flag leaf width was close to 3:1, which indicated that *nal8* is a recessive single gene mutation. A map-based cloning strategy was used to identify the *NAL8* candidate gene. Flag leaf width was the parameter that we used to identify wild type, heterozygous, and homozygous mutant plants. The *NAL8* locus was originally mapped to the short arm of chromosome 7 near the centromere, between marker loci 07–30,610 and 07–52,402. Due to the centromere effect (reduced recombination), we used 10,447 F_2_ plants and developed six different mapping markers, and were able to narrow the mapped region to a 72 kb interval between marker loci NAL8-Q and RM5499 (Fig. 2a). The interval was found to contain 12 open reading frames (ORFs), most of which are transposons and retrotransposons. We sequenced the ORFs and found a single G to A nucleotide transition at position 682 in the candidate gene LOC_Os07g15880. According to the MSU rice annotation database, the candidate gene LOC_Os07g15880 is predicted to encode a mitochondrial prohibitin complex protein 2α subunit. Analysis of the mutant and wild-type sequences suggested that this mutation causes an alanine to a threonine change at amino acid A228T. We then used the SWISS-MODEL website (https://swissmodel.expasy.org/) to predict the protein structure of the NAL8 candidate protein. SWISS-MODEL predicts that the NAL8 candidate protein is capable of forming a homotrimer (Fig. 2b). When the amino acid at position 228 is changed from an alanine to a threonine, a peptide loop in the homotrimer structure is altered. In addition, the predicted protein global quality estimate also changed. We further compared the NAL8 protein with NAL8 homolog proteins from other monocot and dicot plants; NAL8, contains an SPFH prohibitin domain, and the alanine at position 228 appears to be a highly conserved residue (Fig. 2c and Additional file 2: Figure S2). A phylogenetic analysis shows that this protein is conserved in eukaryotes, including mammals, and that NAL8 is closely associated with proteins from other species of *Oryza* in a clade that contains only proteins from monocots (Additional file 3: Figure S3).

To determine whether the mutation in the *NAL8* candidate gene is responsible for the observed mutant phenotype, we performed a complementation test with a DNA fragment from the wild-type line TQ containing the putative promoter region, the entire ORF, and the putative 3′ untranslated region (3′-UTR) of *NAL8*. This fragment was introduced into the *nal8* mutant via *Agrobacterium tumefaciens*-mediated transformation. We found that the transgenic complementation lines rescued the growth defects in the *nal8* plants, including recovery of normal leaf width (Fig. 2d, e, f). In addition, we compared the panicles and grain sizes in plants of TQ, the *nal8* mutant, and two individual complementation transgenic lines. The transgenic lines successfully rescued the reduced spikelet number caused by the *NAL8*^*A228T*^ mutation (Additional file 4: Figure S4A, B). Other agronomic traits, such as plant height and thousand seed weight, were also restored to wild-type levels in the complemented transgenic lines compared with *nal8* (Additional file 4: Figure S4C-H). These results confirm that LOC_Os07g15880 is the *NAL8* gene.

### *NAL8* transgenic plants display consistent phenotypes with *nal8* mutant

To further explore the phenotypes of mutations in the *NAL8* gene, we used the CRISPR/Cas9 genome editing system to generate *NAL8* knockout transgenic plants in *japonica* rice variety ‘Zhonghua-11’ (ZH11). We obtained two independent CRISPR/Cas9 genome editing knockout lines, and nucleotide sequence alignments showed that one base pair was deleted in *NAL8*^*CRISPR*^ #1, and two base pairs were deleted and one base pair was inserted in *NAL8*^*CRISPR*^ #2 (Additional file 5: Figure S5). Both mutations result in predicted translational frame shifts that cause missense mutations in the NAL8 protein. The knockout plants displayed similar phenotypes to the *nal8* mutant, including a narrow flag leaf, reduced plant height, and fewer spikelets (Additional file 6: Figure S6A-C). Other agronomic traits, such as thousand seed weight, grain size, and tiller number were also significantly different compared with wild type ZH11 (Additional file 6: Figure S6D-K). These results suggest that *NAL8* is responsible for these phenotypes in all rice subspecies. Consistent with the phenotypes of the *NAL8*^*CRISPR*^ transgenic plants, we constructed RNAi-silenced transgenic lines in the TQ background. The two independently-derived *NAL8*-silenced lines displayed similar reductions in flag leaf width, together with reduced spikelet number and plant height (Additional file 7: Figure S7). These results indicate that the changes in NAL8 protein amounts and protein structure led to similar plant developmental defects. We also constructed the overexpressing transgenic lines in the ZH11 background in which *NAL8* is driven by the CaMV 35S promoter. The *NAL8*-overexpressing plants did not show obvious differences in plant height and flag leaf width compared with wild-type ZH11 plants (Additional file 8: Figure S8). In contrast, the *NAL8*^*OE*^ transgenic lines showed reduced flag leaf length and thousand seed weight, with slightly higher spikelet numbers than in ZH11 plants. These results show that over-expression of the *NAL8* gene in transgenic rice plants did not have significant effects on plant development.

We also carefully examined grain shape under the stereo microscope. The grains were smaller because their width was reduced in the *NAL8*^*CRISPR*^ transgenic lines (Additional file 6: Figure S6H; Additional file 9: Figure S9A), while the *NAL8*^*RNAi*^ transgenic lines had grains that were narrower but longer (Additional file 9: Figure S9B). The *NAL8*^*OE*^ transgenic lines had smaller grains compared with ZH11 (Additional file 9: Figure S9C), and the *NAL8*^*com*^ transgenic restored the longer grain length and thinner grain width that is characteristic of the *nal8* mutants (Additional file 9: Figure S9D). Taken together, the *NAL8* gene in the *indica* variety ‘TeQing’ is responsible for leaf width, spikelet number and grain size in rice.

### *NAL8* encodes a prohibitin complex 2α subunit and is essential for chloroplastic development

The *NAL8* gene (LOC_Os07g15880) is predicted to encode a prohibitin complex 2α subunit. The prohibitin complex has been well studied in yeast, *C. elegans*, and mammals [23]. Previous studies have suggested that the prohibitin complex assembles into a ring-like macromolecular structure at the inner mitochondrial membrane and is involved in multiple cellular processes. In *Arabidopsis*, PHB3 is a nucleo-mitochondrial dual-localized protein that maintains genome integrity and cell proliferation in the root meristem through *MINICHROMOSOME MAINTENANCE 2* (*MCM2*) [31]. Another recent study also showed that PHB3 is localized in the chloroplasts [30]. Considering that PHB3 forms complex with other PHBs, we inferred that NAL8 is localized in mitochondria and chloroplasts. We further assayed the expression patterns of *NAL8* using qRT-PCR and GUS staining methods, and found that the gene is globally expressed in almost all tissues, a finding that is consistent with a general function in cell surface migration, cell cycle regulation, mitochondrial respiration, cell senescence, and cell death (Additional file 10: Figure S10A-I) [26, 35–38].

**Fig. 3:**
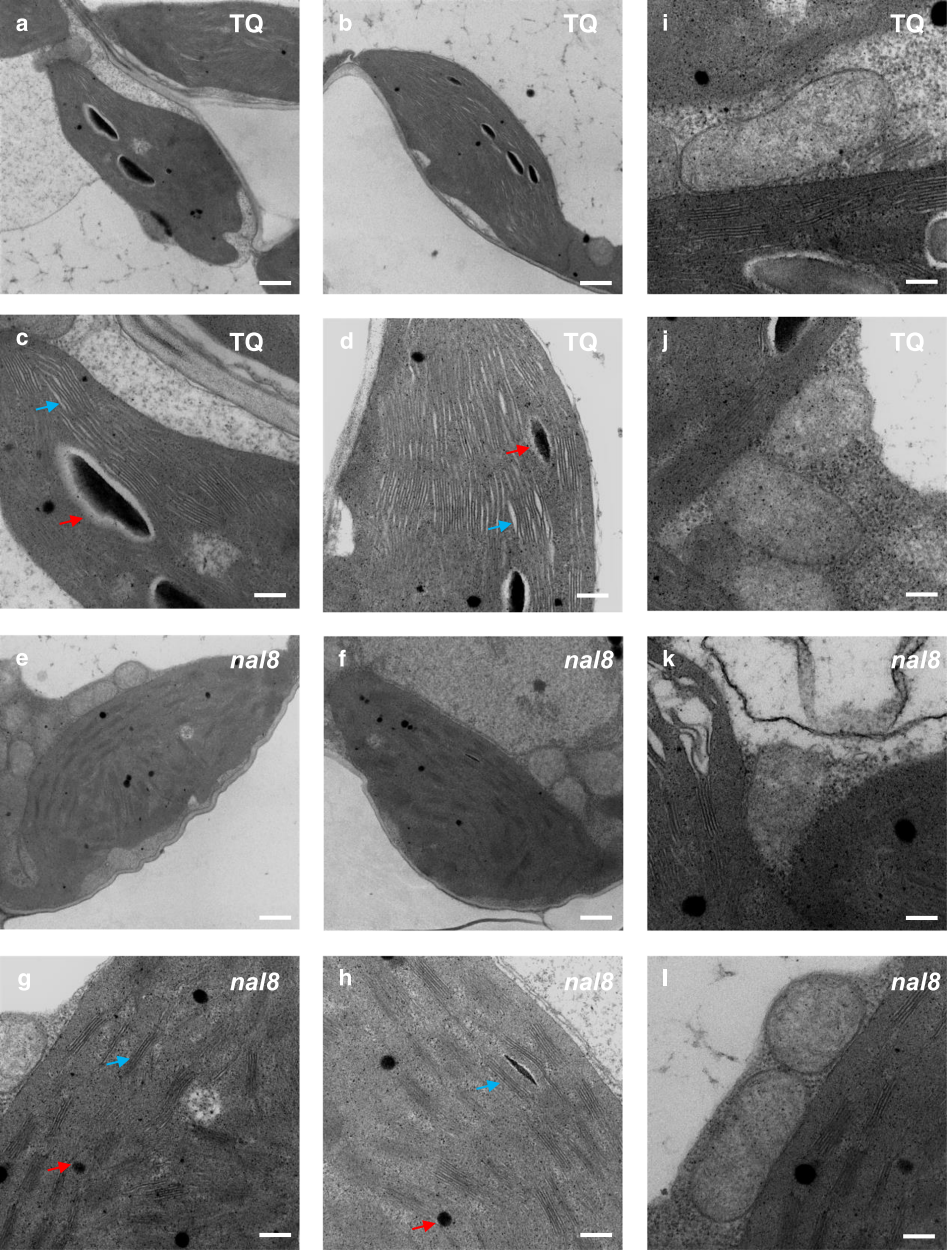
Leaf cell chloroplasts of the *nal8* mutant have smaller plastoglobules and aberrant thylakoids. **a**, **b** Transmission electron microscope images of intact chloroplastic ultrastructure of *indica* line TQ. Scale bar = 1 μm. **c**, **d** Enlarged images from **a** and **b** showing details of the ultrastructure of line TQ chloroplasts. Blue arrows indicate the continuous thylakoids, and red arrows indicate the plastoglobules. Scale bar = 250 nm. **e**, **f** Chloroplastic ultrastructure of the *nal8* mutant line. Scale bar = 1 μm. **g**, **h** Enlarged images of **e** and **f** showing details of the ultrastructure of chloroplasts from the *nal8* mutant. Blue arrows indicate the thylakoids, and red arrows indicate the plastoglobules. Scale bar = 250 nm. **i**-**l** Ultrastructure of intact mitochondria from line TQ (**i**, **j**) and the *nal8* mutant (**k**, **l**). Scale bar = 250 nm

To further explore the changes in ultrastructure in the *nal8* mutants, we observed the chloroplasts and mitochondria in wild-type TQ and *nal8* mutant cells by transmission electron microscopy (TEM). In TQ, the chloroplastic images showed intact double membrane structures and continuous thylakoids, with normal sized starch granules and plastoglobules (Fig. 3a, b, c, d). However, the sizes of the grana and plastoglobules were significantly reduced in the chloroplasts of the *nal8* mutants (Fig. 3e, f, g, h). Furthermore, the grana sacs were thinner, darker, and discontinuous in *nal8* chloroplasts, indicating that the structure of the thylakoids in the *nal8* mutant is damaged and that photosynthetic efficiency could be diminished. We also observed the mitochondria in TQ and *nal8* cells. However, due to the low resolution of the mitochondria, we were not able to obtain clear images of mitochondrial ultrastructure (Fig. 3i, j, k, l). Taken together, our results show that *NAL8* is involved in morphogenesis in normal chloroplasts.

### *NAL8* influences cell proliferation through modulating cell cycle-related gene expression

**Fig. 4:**
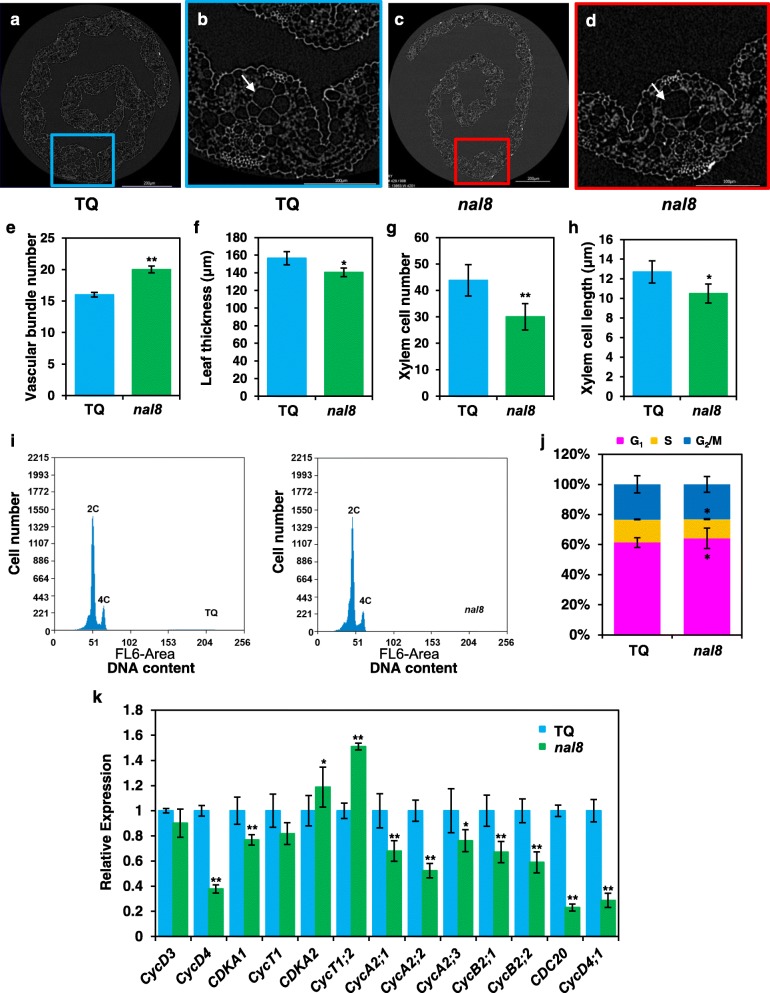
*NAL8* contributes to localized cell proliferation and cell size. **a** The visual splices of leave cross-section surface of TQ. Scale bar = 200 μm. **b** Enlargement of the region outlined in blue in **a** showing the TQ vascular bundles. Scale bar = 100 μm. **c** The visual splices of leave cross-section surface of *nal8* mutants. Scale bar = 200 μm. **d** Enlargement of the region outlined in red in **b** showing the vascular bundles in the *nal8* mutant. Scale bar = 100 μm. The arrows indicate the cell division aberration in the *nal8* mutant. The visual splices were performed by X-Ray microscope from Zeiss company. **e**-**h** Statistical analyses of vascular bundle number (**e**), leaf thickness (**f**), xylem cell number (**g**), and xylem cell length (**h**) in TQ and the *nal8* mutant based on X-ray microscope observation (*n* = 3 leaves). Values are given as the mean ± SD. **P < 0.01 compared with the TQ control using Student’s *t*-test. **i** Flow cytometry analysis of root tip (1–3 cm) cells from TQ and the *nal8* mutant. The two peaks represent 2C and 4C nuclei, respectively. **j** Percentage of cells at different phases of the cell cycle in young root tip cells (*n* = 10 pooled tissues, and three young root tips from different plants per pool). The G_1_, S, and G_2_/M phases are shown by colored boxes. **k** Relative expression levels of the genes involved in the cell cycle in TQ and the *nal8* mutant. The *UBQ5* gene was used as an internal reference to normalize the gene expression data. Values in **e**-**h**, **j**, **k** are given as the mean ± SD. *P < 0.05; **P < 0.01 compared with the TQ control using Student’s *t*-test

To further explore the narrow-leaf phenotypes of the *nal8* mutants at the subcellular level, we used X-ray microscopy (XRM) to observe the cross-section surface of young leaves from fresh samples of TQ and *nal8* seedlings (Fig. 4a, b, c, d). Observation of transverse sections indicated that the number of leaf vascular bundles is increased in *nal8* compared with TQ (Fig. 4e). In addition, the *nal8* mutant also displayed decreased leaf thickness, and reduced cell number and size in the leaf xylem elements (Fig. 4f, g, h), suggesting that cell division and cell elongation are both affected by the mutated form of the NAL8 protein. We then performed flow cytometry to analyze the cell division rate in root tips. We found that the number of cells with 2C DNA content in the G_1_ phase was higher in *nal8* than in TQ. There were also fewer S phase cells in *nal8* than in TQ, and the percentage of G_2_/M phase cells with 4C DNA content was almost identical in *nal8* root tips (Fig. 4i, j). These data indicate that the mutated NAL8 protein disrupts cell proliferation, which leads to severely altered plant morphology. Our conclusion was further supported by qRT-PCR of cell cycle-related genes in TQ and the *nal8* mutant. The expression levels of most of these genes were significantly down-regulated in the *nal8* mutant (Fig. 4k). In summary, our data suggest that the mutated of NAL8 protein causes a reduction in the expression levels of cell cycle-related genes, and the resulting effects on the cell division profoundly alter leaf width and spikelet structure in the *nal8* mutant plants.

### *NAL8* is involved in the maintenance of chloroplastic and mitochondrial structures, and regulates multiple hormone signaling pathways

Previous studies have shown that prohibitins regulate salicylic acid biosynthesis [30]. We suspected that NAL8 functions as a prohibitin complex subunit and is also involved in hormone signaling regulation networks. Therefore, we measured the levels of multiple endogenous hormones in TQ and *nal8* and found that two major plant development-related hormones, auxin and cytokinin, were significantly altered. The indole-3-acetic acid (IAA) levels were higher in *nal8* plants than in TQ, while levels of the inactivated form methyl-IAA (Me-IAA) showed the opposite trend (Additional file 11: Figure S11A, B). These data indicate that auxin signaling is over-activated in the *nal8* mutant compared to TQ. Consistent with the observed phenotypes, the cytokinin hormones isopentenyladenine (iP) and trans-zeatin (tZ) were significantly reduced in *nal8* compared to TQ, which tends to support the reduced cell division status in *nal8* (Additional file 11: Figure S11C, D). The levels of other hormones, such as jasmonic acid (JA), salicylic acid (SA), indole-3-carboxylic acid (ICA), and cis-zeatin (cZ) were also measured in TQ and *nal8* with no significant differences found (Additional file 11: Figure S11E-H).

We further performed RNA-seq in 7-day-old TQ and *nal8* seedling. The experimental group and the control group both consisted of three biological repeats. Sample correlation and principal component analysis (PCA) showed that these two groups were widely separated on the first component axes (PC1), while the repeats within each group were highly correlated (Additional file 12: Figure S12A, B). A volcano plot showed that there were 8876 differentially-expressed genes (DEGs) (5477 and 3399 were up- and down-regulated, respectively) between the mutant group and the control group (Additional file 12: Figure S12C). All DEGs are listed in (Additional file 17: Table S1) in the Supporting Information. A correlation plot also showed that the up-regulated genes were more scattered and that there were more of them compared with the down-regulated genes (Additional file 12: Figure S12D). Kyoto Encyclopedia of Genes and Genomes (KEGG) pathway analysis indicated that the DEGs were significantly enriched in multiple amino acid biosynthetic and metabolic pathways, together with ribosome components in the *nal8* mutants compared with the wild type (Additional file 13: Figure S13A). We also performed Gene Ontology (GO) enrichment of the DEGs, and found that most of them were related to the basic function of the ribosome, mitochondria and chloroplasts (Additional file 13: Figure S13B). All of these results strongly suggest that *NAL8* functions in translation and organelle structural stability.

**Fig. 5:**
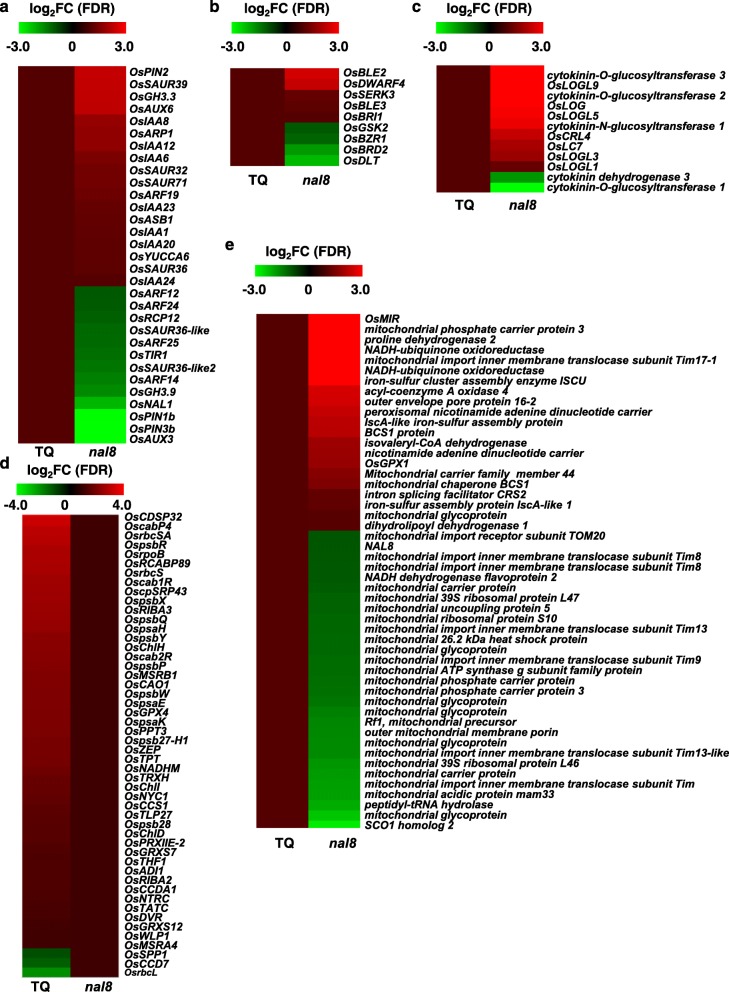
*NAL8* contributes to multiple hormone signaling and development of mitochondria and chloroplasts. **a**-**e** Heat maps show that expression of auxin-related genes (**a**), brassinosteriod-related genes (**b**), cytokinin-related genes (**c)**, chloroplast-related genes (**d**) and mitochondria-related genes (**e**) is significantly different in TQ and the *nal8* mutant as determined from the RNA-seq data. Log_2_FC is the logarithm of two-fold enrichment of relative gene expression

We therefore constructed heatmaps of hormone-related genes and organelle-related genes in TQ and *nal8*. The heatmap for auxin, brassinosteroid (BR), and cytokinin signaling showed that the expression patterns for many phytohormone signaling and transduction genes are changed in the *nal8* mutant, which indicates that *NAL8* may affect plant development by interfering with plant hormone signaling transduction (Fig. 5a, b, c). Moreover, expression of the fundamental chloroplast house-keeping genes in *nal8* was reduced, leading to aberrant chloroplastic ultrastructure and decreased photosynthetic efficiency (Fig. 5d). Changes also occurred around mitochondria-related genes, causing weakened respiration in the *nal8* mutant (Fig. 5e). To further verify our RNA-seq findings, we performed qRT-PCR assays to quantify the relative expression of key respiration-related genes relative expression in TQ and *nal8* (Additional file 14: Figure S14A). The results showed that genes for the mitochondrial precursors of *ubiquinol oxidase 1a* (*AOX1a*) and *ubiquinol oxidase 1c* (*AOX1c*), and also the NAD(P) H dehydrogenases (*NDA* and *NDB3*) in *nal8* had higher levels of expression than in TQ, suggesting that expressions of these genes in mitochondria were possibly increased to compensate for the loss of NAL8 function. We performed qRT-PCR assays towards chloroplast-related genes. Consistent with RNA-seq results, almost all chloroplast-related gene expressions are decreased in *nal8* mutant (Fig. 5d) (Additional file 14: Figure S14B), indicating the chloroplast functions are severely damaged in *nal8* mutant. We also performed qRT-PCR assays of BR-related genes, which showed that the relative expression of many BR transduction genes was altered, suggesting that *NAL8* also be involved in BR signaling (Additional file 14: Figure S14C). Taken together, the endogenous hormone levels and RNA-seq results indicate that the *NAL8* gene is involved in signaling for multiple plant hormones as well as normal mitochondria and chloroplasts function.

### *NAL8* alters protein composition in mitochondria and chloroplasts and involves normal nitric oxide maintenance

**Fig. 6:**
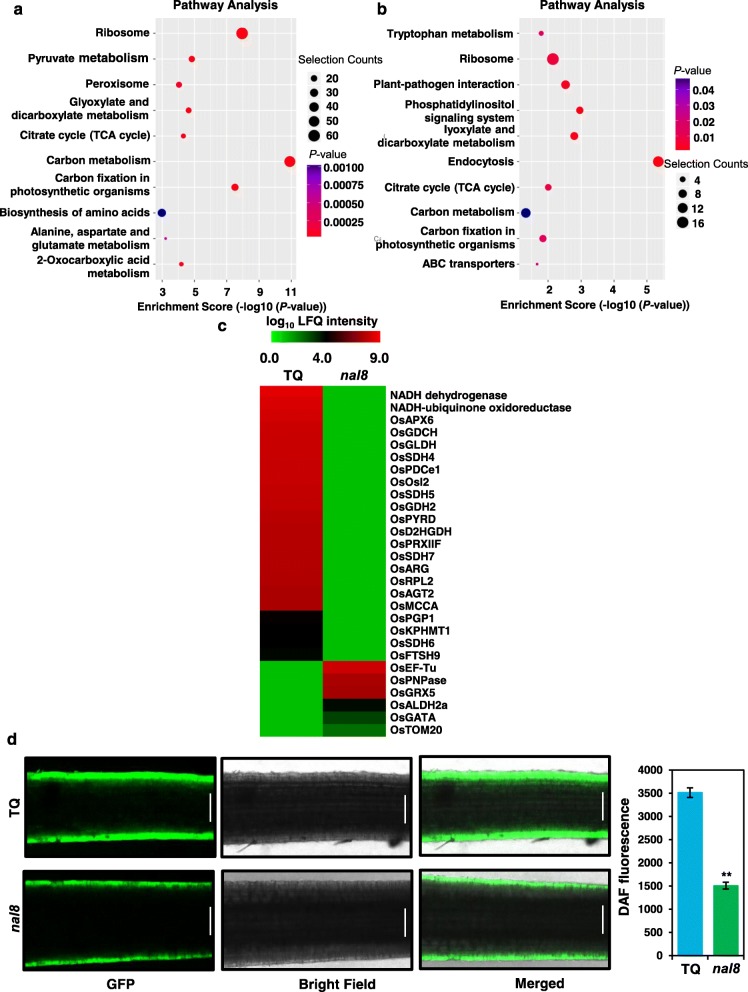
*NAL8* is involved in mitochondria and chloroplasts stability, and maintenance of normal NO levels in rice roots. **a**, **b** KEGG pathway enrichment analysis of proteomic data from TQ and *nal8* for the up-regulated proteins (**a**) and the down-regulated proteins (**b**). The y-axes show the KEGG pathways, and the x-axes show the enrichment scores for all differentially expressed genes. The circle size is the relative size of the selection gene amounts. -log_10_ of the adjusted *P* value is shown on the x-axes. (false discovery rate) *FDR* < 0.01. **c** A heatmap showing the mitochondrial- and chloroplast-related proteins enriched in TQ and the *nal8* mutant. Log_10_ transformed and quantile-normalized LFQ intensities are shown. **d** Rice seedlings were grown in liquid medium for 7 days for NO detection using 10 mM (micro molar per liter) DAF-FM diacetate, a fluorescent probe for NO. Scale bar = 0.5 mm. Similar results were obtained for all three biological replicates. Values represent the mean ± SD. **P < 0.01 compared with TQ using Student’s *t*-test

To determine exactly how NAL8 alters mitochondria and chloroplasts, we performed a proteomic analysis on TQ and the *nal8* mutant. We used young seedlings of TQ and the *nal8* mutant that were grown under conditions similar to those used in the RNA-seq assays. We identified 570 different proteins were up-regulated and 298 different proteins that were down-regulated in TQ compared with the *nal8* mutant (Additional file 15: Figure S15A). All proteins identified by proteomics and the differential protein results are given in (Additional file 18: Tables S2 and Additional file 19: Table S3). It was obvious that the differentially-occurring proteins were scattered widely in the volcano plot, indicating that the *nal8* mutation causes tremendous changes in the expression of these proteins (Additional file 15: Figure S15B). KEGG pathway analysis in TQ showed that the up-regulated proteins were enriched in ribosome, carbon metabolism, tricarboxylic acid cycle (TCA cycle), and pyruvate metabolism pathways (Fig. 6a). Consistent with the up-regulated protein enrichment results, the down-regulated proteins were enriched in ribosome, carbon metabolism, TCA cycle, and “carbon fixation in photosynthetic organisms” pathways (Fig. 6b). These pathways are mainly present in mitochondria and chloroplasts, confirming that NAL8 is involved in protein homeostasis in mitochondria and chloroplasts. We also selected several differentially expressed mitochondrial proteins from the proteomics results and found that the expression of many key mitochondrial protein is significantly reduced in the *nal8* mutant (Fig. 6c). A previous study showed that mutation of PHB3 significantly reduced Nitric Oxide (NO) transport in *Arabidopsis* [29]. We also used the fluorescent dye DAF-FM DA to quantify NO content in TQ and *nal8* mutant roots. The results suggest that the NO content is reduced in the *nal8* mutant roots (Fig. 6d), supporting the hypothesis that NAL8, as prohibitin 2α, is responsible for NO accumulation in the roots, similar to the results for the *phb3* mutant in *Arabidopsis*. Taken together, these results show that NAL8 and other prohibitin proteins might function as scaffold proteins to stabilize mitochondrial and chloroplastic structure to maintain protein homeostasis and NO accumulation associated with cell metabolism and photosynthesis-related cell activities that contribute to normal plant growth and development.

## Discussion

In summary, the results suggest that the molecular chaperone NAL8, a member of the prohibitin complex, plays crucial roles in leaf width and spikelet number by modulating the stability of mitochondria and chloroplasts in rice. A previous study in rice showed that OsPHB1 is phosphorylated in response to calyculin A, an inducer of defense responses [34]. In *Arabidopsis*, *PHB3*, a homolog of *NAL8*, is involved in multiple functions linking SA signaling, H_2_O_2_-mediated NO and ROS signaling, ethylene signaling and even DNA integrity and proliferation in the nucleus [28–32]. Our results provide new leaf morphology and grain number phenotypes caused by a mutation in the *NAL8* gene (Fig. 1), suggesting similar molecular mechanisms for the action of PHBs in plants.

Leaf morphology is strongly associated with chloroplastic and mitochondrial development. The morphogenesis of leaf development contains three stages: The initiation of leaf primordium, the establishment of leaf polarity, and the development of leaf margin [39]. Many yellow leaf mutants and aberrant leaf shape mutants have been isolated and characterized [4], and these phenotypes are due to mutations in genes involved in phytohormone metabolism, cell division, number of veins ionic homeostasis and epigenetic manner. Prohibitins contribute to multiple leaf phenotypes in *Arabidopsis*; however, in cereal crops, the function of prohibitins has been unclear until now. Moreover, few genes that regulates leaf morphology and grain numbers has been discovered. In this study, we identified *nal8*, a rice mutant with narrow leaf and reduced grain number (Fig. 1), and generated the CRISPR/Cas9 knockout mutants and RNAi lines to observe the related phenotypes. Although the *nal8* mutant, RNAi and CRISPR/Cas9 knockout transgenic lines displayed similar narrow leaf morphology and reduced grain number phenotypes (Fig. 1b and c; Additional file 6: Figure S6B and C; Additional file 7: Figure S7B and C), the other traits, such as leaf length, grain length and width, were not exactly consistent changes. Because the *nal8* mutant and RNAi lines were in TQ (*indica* variety) backgrounds, and the CRISPR/Cas9 knockout lines were in ZH11 (*japonica* variety) background, we suspected that the rice variety backgrounds also affect on these traits. Previous researches revealed that dysfunctional mitochondrial and chloroplastic structure affect leaf development in *Arabidopsis*. SLO3, a pentatricopeptide repeat protein that contributes to intron removal of *NAD7* encoding an NADH dehydrogenase subunit 7 in mitochondria, also interacts with auxin signaling pathways to regulate the boundary of root apical meristem and leaf shape in *Arabidopsis* [40]. *CRUMPLED LEAF* (*CRL*) gene, encoding a protein localized in the outer membrane of plastids, affects the normal cell division, cell differentiation and plastid division in *Arabidopsis*. Recent studies revealed that in *crl* mutants caused chloroplastic dysfunction and multiple cell cycle progression defects in *Arabidopsis* [41, 42]. The *Arabidopsis ARC5* and *ARC6*, encoding a cyto-plasmic dynamin-related protein and an inner envelope transmembrane protein respectively, contribute to plastid morphology in leaf epidermal pavement cells and stomatal guard cells [43]. These results indicated that dysfunction in mitochondria and chloroplasts disrupted normal cell division leading to leaf development. In our study, we found that organelles (chloroplasts and mitochondria) are altered in *nal8* mutants (Fig. 3), and cell division processes are also affected by the mutation in *NAL8* (Fig. 4), supporting the notion that *PHB3* regulates cell proliferation in the root meristem through MINICHROMOSOME MAINTENANCE 2 [32]. It is possible that other OsPHBs also contribute to cell division processes, leading to normal respiration and photosynthesis in mitochondria and chloroplasts. Based on a phylogenetic tree constructed from alignments of OsPHB and AtPHB protein sequences, the well-studied AtPHB3 protein belongs to the type-I prohibitin class, and NAL8 is a type-II prohibitin (Additional file 16: Figure S16) and its function has yet to be reported in plants. These results suggest potential functional differentiation of the proteins in the two PHB subgroups. In addition, we used RNA-seq and proteomics tools to reveal the changes in transcription and translation levels of genes/proteins in TQ and the *nal8* mutant (Figs. 5 and 6), indicating that prohibitin complexes may play important roles in plant development. Phytohormones, such as auxin and gibberellins, have been shown to play essential roles in the determination of leaf size [4]. Our results show that the auxin level in *nal8* is higher than in the TQ (Additional file 11: Figure S11A), suggesting a potential compensation effect of the auxin signaling pathway in *nal8* mutant plants. Interestingly, the cytokinin levels in *nal8* were considerably reduced, supporting the notion that cytokinin and auxin signaling are antagonistic. In rice, the *OsCKX2* gene encodes a cytokinin oxidase [11], and mutation of *OsCKX2* results in increased cytokinin levels in apical meristem tissues, leading to increased spikelet numbers and grain number per panicle. Moreover, apical dominance associated with grain number is regulated by auxin and cytokinin in rice. The KNOX family transcription factor shoot meristem-less (STM) and homeodomain-containing transcription factor WUSCHEL (WUS) coordinate shoot meristem development with CLAVATA (CLV) [44, 45]. Overexpressing *KNOX* genes can rapidly induce the accumulation of *IPT*, a gene that encodes a key rate-limiting enzyme in the cytokinin biosynthesis pathway [46], which suggest that cytokinin plays vital roles in shoot meristem development. Moreover, previous studies showed that the chlorophyll content from in vitro apple leaves was positively related with cytokinin treatment [47], and the *CYTOKININ-HYPERSENSITIVE* genes *CKH1* and *CKH2* of *Arabidopsis* negatively regulated the cytokinin-signaling for cell division and chloroplastic development [48], supporting the notion that chloroplast function is essential for cell division.

## Conclusions

In conclusion, the results of our study suggest that prohibitin family proteins in rice are required for internal homeostasis of mitochondrial and chloroplastic proteins. Our findings shed new light on the function of small molecular chaperones as scaffold proteins involved in the formation of vital organelle structure and cell division processes. Further identification of additional OsPHB family members that target leaf morphology and grain number in rice via the underlying processes of cell division and proliferation will assist modern rice breeding programs by balancing plant abiotic stress capabilities with high grain yield.

## Methods

### Plant materials and growth conditions

The *nal8* mutant was obtained from EMS treatment of the *Orzya sativa* ssp. *indica* rice variety ‘TeQing’. The M_1_-generation *nal8* mutant was crossed back to TQ to obtain the mutation in an isogenic background. TQ and the *japonica* rice variety ‘Zhonghua-11’ were used for plant transformation. All plants were cultivated in Shanghai and Hainan under natural growth conditions.

### Map-based cloning of *NAL8*

The *japonica* variety ‘Jiahua-1’ was crossed with the *nal8* mutant to obtain F_1_ plants. The F_1_ was self-pollinated to produce an F_2_ mapping population. To fine-map the *NAL8* locus, we designed several new molecular markers from predicted SSRs (Simple Sequence Repeats) and InDels (Insertion and Deletion). *NAL8* was originally mapped to a 72 kb (kilo base pairs) region near the centromere on the short arm of chromosome 7 using 10,447 F_2_ plants. All DNA fragments shown in Figure 2a were amplified from both *nal8* and ‘Jiahua-1’ by PCR for map-based cloning. A 2X PCR mix was used to amplify the DNA fragments (Tiangen #KT207). Primer sequences are given in (Additional file 20: Table S4).

### Plasmid construction and plant transformation

To perform the genetic complementation assay, we cloned the full-length *NAL8* genomic DNA sequencing consisting of 2.5 kb DNA upstream of the NAL8 start codon and 500 bp DNA downstream into the pCAMBIA-1300 binary vector. The 2.5 kb DNA fragment containing the *NAL8* promoter region was then cloned into the pCAMBIA-1300-GUSplus vector to generate *NAL8*_*Pr*o_::GUS for GUS staining experiments. To generate the CRISPR/Cas9 knockout transgenic plants, we used the CRISPR-GE website (http://skl.scau.edu.cn/) to design gRNA targets and identify the mutated positions [49]. To generate the overexpression transgenic constructs, the full length *NAL8* cDNA was amplified from TQ RNA and cloned into the plant binary vector pCAMBIA 1301 under control of the CaMV 35S promoter. To generate the *NAL8* knockdown constructs, we designed the target mimic microRNAs using the WMD3 website (http://wmd3.weigelworld.org/cgi-bin/webapp.cgi). The target DNA fragments were then inserted into the pCAMBIA-1306-35SN vector. *Agrobacterium tumefaciens*-mediated transformation of rice was performed as previously described [50], using the EHA105 strain for rice transformation. The identities of all DNA constructs were confirmed by sequencing, and all positive transgenic plants and negative controls were selected by PCR amplification of the hygromycin resistance gene. The results of qRT-PCR assays of *NAL8* gene expression were also used to evaluate the over-expression and knockdown transgenic lines. To confirm the introduced mutations in the CRISPR/Cas9 knockout plants, we designed specific primers for sequencing the mutated nucleotide positions. All plasmid constructs in this study were generated using NEBuilder HIFI DNA Assembly Master Mix (New England Biolabs, catalog#2621 L). The PCR primer pair sequences were given in (Additional file 20: Table S4).

### GUS staining

GUS enzyme staining of *NAL8*_*Pro*_::GUS transgenic plants was performed as described previously [51]. Samples were obtained in the field at the reproductive stage, and were incubated in GUS staining solution at 37 °C overnight. The samples were then washed in 75% ethanol to remove the chlorophyll, and were imaged with a Leica model M205C stereo microscope.

### X-ray microscopic observation

TQ and *nal8* seedlings were grown in an illuminated chamber for 7 days after germination. Samples were collected and fixed in FAA (50% ethanol, 5% glacial acetic acid, 5% formaldehyde) for 12 h at 4 °C. After the tissue was dehydrated in a graded ethanol series, the samples were thoroughly desiccated in an automated Critical Point Dryer (Leica EM CPD300). Samples were observed with a Zeiss Xradia 520 Versa X-Ray Microscope. XM 3DViewer was used to generate the visual image slices.

### RNA extraction and quantitative real-time PCR (qRT-PCR)

Total RNA was extracted from plant tissue samples using the E.Z.N.A.® Total RNA Kit (Omega Bio-Tek, #R6834–1). The RNA was quantified with a GEN5 microplate reader (Bio-Tek). Complementary DNA (cDNA) synthesis was performed using ReverTra Ace® qPCR RT Master Mix with gDNA remover (Toyobo #FSQ-301). qRT-PCR assays were performed on the ABI 7300 Real-Time PCR System (Applied Biosystems) using the Fast Start Universal SYBR Green Master Mix with ROX. The *UBQ5* gene expression was used as internal reference to normalize the gene expression data. The 2^-ΔΔCT^ method was used to analyze the expression data [52]. Nucleotide sequences of the PCR primer pairs used in the qRT-PCR assays are given in (Additional file 20: Table S4).

### Nucleus isolation and ploidy determination

Isolation of cell nuclei and ploidy measurements were performed as described previously [53]. The root tips of seedlings grown in glass tubes for 3 days after germination were chopped, and the nuclei were isolated and stained in nuclear isolation and staining solution (NPE Systems #7216). The suspension was filtered through a 40 μm nylon filter (Thermofisher #352340), and flow cytometry was performed on a Beckman Moflo cell sorter. Approximately 10,000 nuclei per sample were analyzed. The relative proportions of G_1_, S, and G_2_/M phase nuclei were calculated by FCS express 4 software.

### Observation of chloroplastic and mitochondrial ultrastructure

We used transmission electron microscopy (TEM) to observe the ultrastructure of chloroplasts and mitochondria as previously described [54]. Young seedling leaves of TQ and *nal8* were chopped into 2 mm × 4 mm pieces and fixed in 2% glutaraldehyde solution for 2 h. Then tissue pieces were first washed in 0.1 M PBS and then 5–8 times in distilled water. After dehydration in a graded ethanol series, the samples were soaked briefly in two changes of propylene oxide. Sample were then transferred to propylene oxide and Quetol 812 resin, covered with aluminum foil, and incubated overnight, after which they were embedded in Quetol 812 resin in a plastic flat embedding mold for 2 days. The tissue samples were then sectioned with a diamond knife on an ultramicrotome (70 to 100 nm) and transferred to a copper grid for observation using a Hitachi H-7650 transmission electron microscope.

### RNA-sequencing

RNA-seq was performed by Biomarker Technologies Corporation (Beijing, China). We used young seedlings of three independent lines of TQ and the *nal8* mutant to generate the RNA libraries for transcriptome sequencing. The NEBNext Ultra™ RNA Library Prep Kit (NEB) was used for Illumina high-throughput sequencing. All the raw data in fastq format was processed using perl scripts. The Q20 and Q30 percentages, the GC-content, and the relative level of sequence duplication were also calculated from the clean data. Pearson’s Correlation Coefficient was conducted to evaluate the repetition correlation in the two samples [55]. Differential expression analysis of two conditions/groups was performed using DEseq [56]. Genes with an adjusted *P*-value < 0.01 identified by DEseq were considered to be differentially expressed. GO enrichment analysis [57] and KEGG pathway enrichment analysis [58] was based on Wallenius non-central hyper-geometric distribution, and KOBAS software was used to test the statistical enrichment of DEGs in the KEGG pathways [59]. Heatmaps were generated using MeV software. We have uploaded the RNA-seq into the NCBI, the NCBI SRA accession number is PRJNA557518.

### Proteomics analysis

The proteomics analysis and MS service were performed by Cloud-Seq Biotech Ltd. Co. (Shanghai, China). Samples were collected from three independent young seedlings of both inbred line TQ and the *nal8* mutant. Protein samples were digested as previously described [60]. After digestion, the protein fragments were separated by capillary high performance liquid chromatography (μHPLC). The MS machine type is Q-EXACTIVE (Thermo Fisher Scientific, CA, USA). The peptides and their fragments were collected by a full scan and 12 fragment scans. Raw data was analyzed using MaxQuant 1.6.0.16 software [61]. The label-free quantification (LFQ) was obtained with the MaxQuant algorithm [62]. LFQ values were log10 transformed, and quantile standardized using the limma R package software. To find statistically significant differences between the TQ and the *nal8* mutant, Two-tailed, Student’s *t* test was performed, with *p*-value 0.05 and fold change 2.0 as cutoff [63]. GO enrichment and KEGG pathway analyses were performed similar to the description in the “RNA-sequencing” section above. Heatmaps were generated using MeV software. We have uploaded the proteomic raw data into the iProX [64], the iProX accession number is PXD015063.

### Nitric oxide (NO) detection in roots

Young seedlings of TQ and the *nal8* mutant were cultured on 0.5X Murashige and Skoog (MS) medium for 5 days. The roots were incubated in a 10 μM solution of DAF-FM diacetate overnight. After washing in distilled water, the samples were observed with an Olympus FluoView FV1000 laser scanning confocal microscope.

## Additional files

Additional file 1: **Figure S1.** Morphology and statistical analysis of mature grains and leaf length in TQ and the *nal8* mutant. (A) Phenotypes of mature grains of TQ and the *nal8* mutant. Scale bar = 2 mm. (B) Comparison of the main spikelets from TQ and the *nal8* mutant. Scale bar = 2 cm. (C-E) Statistical analyses of average flag leaf length (*n* = 20 plants) (C), average grain length (*n* = 20 plants) (D) and average grain width (*n* = 20 plants) (E) between TQ and the *nal8* mutant. Values are given as the mean ± SD. *P < 0.05; **P < 0.01 compared with the TQ control using Student’s *t*-test (C-E). (PDF 57 kb)

Additional file 2: **Figure S2.** Amino acid sequence alignment of homologous proteins to rice NAL8. The conserved alanine residue at position 228 is enclosed in a red box to show the site of the mutation in the *nal8* mutant. The asterisks indicate the highly conserved residues in the proteins from nine plants, three mammals and one insect species. (PDF 668 kb)

Additional file 3: **Figure S3.** Phylogenetic analysis of rice NAL8 and related proteins from plants, animals, and one species of insect. The species names are shown at the ends of the branches. The phylogenetic tree was constructed using the Neighbor-Joining tree method as implemented in MEGA7.0 and embellished with iTOL (http://itol.embl.de/). The numbers shown on each branch indicate protein substitution rate. The NAL8 homologous proteins are highly consistent with the species evolution relationship. (PDF 117 kb)

Additional file 4: **Figure S4.** Comparisons and statistical analysis of TQ, the *nal8* mutant, and *NAL8* complementation transgenic rice lines. (A) Spikelet phenotypes among TQ, *nal8* and the transgenic complementation lines *NAL8*^*com*^#1 and #2. Scale bar = 5 cm. (B) Mature grains of TQ, *nal8* and the complementation lines *NAL8*^*com*^#1 and #2. Scale bar = 2 mm. (C-H) Statistical analyses of average grain width (*n* = 20 plants) (C), average grain length (n = 20 plants) (D), average thousand seed weight (n = 20 plants) (E), average plant height (n = 20 plants) (F), average spikelet number (n = 20 plants) (G) and average flag leaf length (n = 20 plants) (H) in TQ, *nal8* and the transgenic complementation lines *NAL8*^*com*^#1 and #2. Values in C-H are given as the mean ± SD. *P < 0.05; **P < 0.01 compared with the TQ control using Student’s *t*-test. (PDF 80 kb)

Additional file 5: **Figure S5**. Genotyping of the *NAL8*^*CRISPR*^ transgenic knockout lines. Alignment of the DNA sequences of the *NAL8* gene region from the wild-type TQ and the *NAL8*^*CRISPR*^ #1 and #2 lines. Black ellipses show the positions of a 1 bp deletion in the *NAL8*^*CRISPR*^ #1 and a 2 bp deletion in *NAL8*^*CRISPR*^ # 2 sequences (left), and 1 bp insertion in *NAL8*^*CRISPR*^ # 2 (right). The mutations cause translational frame shifts which result in missense mutations in the *NAL8* gene in both transgenic knockout lines. (PDF 49 kb)

Additional file 6: **Figure S6.** Narrow leaf width and reduced spikelet number in the *NAL8* transgenic knockout lines are similar to those in the *nal8* mutant. (A) Plant architecture of the wild-type ZH11 and the *NAL8* knockout lines *NAL8*^*CRISPR*^ #1 and #2 at the reproductive stage. Scale bar = 15 cm. (B) Flag leaves of ZH11 and *NAL8*^*CRISPR*^ #1 and #2. Scale bar = 5 cm. (C) Mature panicles of ZH11 and *NAL8*^*CRISPR*^ #1 and #2. Scale bar = 10 cm. (D-K) Statistical comparisons of the average flag leaf width (*n* = 20 plants) (D), average plant height (n = 20 plants) (E), average spikelet number (n = 20 plants) (F), average thousand seed weight (n = 20 plants) (G), average grain width (n = 20 plants) (H), average grain length (n = 20 plants) (I), average flag leaf length (n = 20 plants) (J) and average tiller number (n = 20 plants) (K) in ZH11 and the *NAL8*^*CRISPR*^ #1 and #2 knockout lines. Values in D-K are given as the mean ± SD. *P < 0.05; **P < 0.01 compared with the ZH11 control using Student’s *t*-test. (PDF 79 kb)

Additional file 7: **Figure S7.** Transgenic *NAL8* gene silenced lines show similar leaf width and spikelet defects to the rice *nal8* mutant. (A) Plant architecture of the wild-type TQ and the transgenic *NAL8* RNAi silenced lines *NAL8*^*RNAi*^ #1 and #2 at the reproductive stage. Scale bar = 15 cm. (B) Flag leaves of TQ and *NAL8*^*RNAi*^ #1 and #2. Scale bar = 5 cm. (C) Mature panicles of ZH11 and *NAL8*^*RNAi*^ #1 and #2. Scale bar = 5 cm. (D-K) Statistical comparisons of average flag leaf width (n = 20 plants) (D), average plant height (n = 20 plants) (E), average spikelet number (n = 20 plants) (F), average thousand seed weight (n = 20 plants) (G), average grain width (n = 20 plants) (H), average grain length (n = 20 plants) (I), average flag leaf length (n = 20 plants) (J) and the relative expression of *NAL8* (*n* = 3 pooled tissues, three plants per pool) (K) in ZH11 and *NAL8*^*RNAi*^ #1 and #2. Values in D-K are given as the mean ± SD. *P < 0.05; **P < 0.01 compared with the TQ control using Student’s *t*-test. The *UBQ5* gene was used as an internal reference to normalize the gene expression data. (PDF 81 kb)

Additional file 8: **Figure S8.** Transgenic rice plants overexpressing *NAL8* show no obvious developmental effects. (A) Plant architecture of the wild-type ZH11 and transgenic *NAL8-*overexpressing lines *NAL8*^*OE*^ #1 and #2 at the reproductive stage. Scale bar = 15 cm. (B) Flag leaves of ZH11 and *NAL8*^*OE*^ #1 and #2. Scale bar = 5 cm. (C) Mature panicles of ZH11 and *NAL8*^*OE*^ #1 and #2. Scale bar = 5 cm. (D-K) Statistical comparisons of average flag leaf width (n = 20 plants) (D), average plant height (n = 20 plants) (E), average spikelet number (n = 20 plants) (F), average thousand seed weight (n = 20 plants) (G), average grain width (n = 20 plants) (H), average grain length (n = 20 plants) (I), average flag leaf length (n = 20 plants) (J) and the relative expression of *NAL8* (n = 3 pooled tissues, three plants per pool) (K) in ZH11 and *NAL8*^*OE*^ #1 and #2. Values in (D-K) are given as the mean ± SD. *P < 0.05; **P < 0.01 compared with the ZH11 control using Student’s *t*-test. The *UBQ5* gene was used as an internal reference to normalize the gene expression data. (PDF 79 kb)

Additional file 9: **Figure S9.** Grain phenotypes of the *NAL8* transgenic lines. (A) Mature rice grains of ZH11 and the transgenic *NAL8*^*OE*^ overexpression lines. Scale bar = 2 mm. (B) Mature rice grains of TQ and the transgenic *NAL8*^*RNAi*^ gene silencing lines. Scale bar = 2 mm. (C) Mature rice grains of ZH11 and the transgenic *NAL8*^*CRISPR*^ transgenic lines. Scale bar = 2 mm. (D) Mature rice grains of TQ, the *nal8* mutant and the transgenic two *NAL8*^*com*^ complementation lines in the *nal8* genetic background. Scale bar = 2 mm. (PDF 118 kb)

Additional file 10: **Figure S10.** Expression profile of *NAL8* in rice tissues and organs*.* (A-H) Histochemical analysis of enzyme activity of the NAL8:GUS fusion protein in the node (A), culm (B), leaf (C), root (D), basal internode (E), spikelet hull (F), young panicle (G), and in transgenic plants expressing the GUS gene under control of the *NAL8* promoter (H). Scale bar = 2 mm. (I) Relative expression of the *NAL8* gene in different rice tissues. The *UBQ5* gene was used as the internal reference to normalize gene expression data. The standard deviations were calculated from three biological replicates. (PDF 83 kb)

Additional file 11: **Figure S11.** Endogenous levels of multiple plant hormones in TQ and the *nal8* mutant. (A-H) Endogenous levels of IAA (A), Me-IAA (B), iP (C), tZ (D), JA (E), SA (F), ICA (G) and cZ (H) in TQ and the *nal8* mutant. Standard deviations were calculated from three biological replicates. (PDF 12 kb)

Additional file 12: **Figure S12.** Comparative RNA-seq analysis between TQ and the *nal8* mutant. (A) Sample correlations among the individual TQ and *nal8* replicates and between TQ and *nal8*. Each sample consisted of three biological replicates. The numbers indicate the pearson’s correlation coefficients for each pair-wise comparison. (B) A PCA map indicates that the TQ and *nal8* samples are widely separated in the RNA-seq analysis. (C) A volcano plot shows that more genes are up-regulated in the *nal8* mutant than are down-regulated compared with TQ. The X-axes shows the logarithmic values of the relative differences in expression. The Y-axes values are the negative logarithms of the significance. Each dot indicates a gene. Red dots and green dots are significantly up-regulated and down-regulated genes, respectively. Black dots are genes in which the expression changes are insignificant. The expression values for the different RNAs were adjusted by FDR < 0.01, and the Fold Change is > 2. (D) Correlation plot of all TQ and *nal8* samples which were used in the RNA-seq analysis. The X- and Y-axes are the logarithmic values of FKPM (fragments per kilobase per million reads). Each dot indicates a gene. The purple line is the coefficient parameter which represents the Pearson’s coefficient of significance, above which the genes are up-regulated, and below which the genes are down-regulated. (PDF 314 kb)

Additional file 13: **Figure S13.** KEGG pathway analysis and GO analysis for genes that are differentially expressed between TQ and the *nal8* mutant**.** (A) All differentially-expressed genes (DEGs) that gave a match in the KEGG (Kyoto Encyclopedia of Genes and Genomes) classification. The numbers and percentages of annotated genes are shown to the right, and the various colors indicate the main KEGG pathway classifications. The x-axes indicates the percentage of annotated genes out of all DEGs. (B) GO (gene ontology) enrichment of DEGs between TQ and *nal8*. The individual GO terms in the three main GO categories are shown on the y-axes, and the x-axes shows the log_10_ of the significance of the corresponding GO terms. (PDF 211 kb)

Additional file 14: **Figure S14.** qRT-PCR analysis of gene expression between TQ and the *nal8* mutant. (A-C) Relative expression levels of mitochondrial genes (A), chloroplast-related genes (B) and brassinosteroid-related genes (C) in TQ and *nal8* as determined by qRT-PCR (*n* = 3 pooled tissues). The *UBQ5* gene was used as an internal reference to normalize the gene expression data. Values represent the mean ± SD. **P < 0.01 compared with the wild-type using Student’s *t*-test. (PDF 46 kb)

Additional file 15: **Figure S15.** Comparative proteomics analysis of TQ and the *nal8* mutant. (A) Venn diagram showing the number of different proteins that are up- and down-regulated in TQ compared with *nal8*. The expression values for the different proteins were adjusted by FDR (false discovery rate). Three biological repeats of both TQ and *nal8* were used to perform the proteomics analysis. (B) Volcano plot showing the distribution of differential protein expression. The x-axes shows the Log_2_ fold-change of the differentially expressed proteins, and the y-axes shows the-log_10_ of the *p*-values of the differences between TQ and *nal8*. The expression values for the different proteins were adjusted by FDR < 0.01, and the Fold Change is > 2. (PDF 227 kb)

Additional file 16: **Figure S16.** Phylogenetic analysis of the PHB proteins from rice and *Arabidopsis*. Phylogenetic analysis of NAL8 and homologous protein sequences from rice (Os) and *Arabidopsis* (At). The gene and/or locus names are shown at the ends of the branches on the phylogenetic tree. The tree was constructed using the Neighbor-Joining method as implemented in MEGA7.0 and embellished with iTOL (http://itol.embl.de/). The numbers shown on each branch indicate protein substitution rate. NAL8 belongs to the type-II class of prohibitins. (PDF 40 kb)

Additional file 17: **Table S1.** All DEGs between TQ and *nal8* in RNA-seq. The numbers are log_2_FC values. (XLSX 780 kb)

Additional file 18: **Table S2.** All proteins identified by proteomics analysis. (XLSX 680 kb)

Additional file 19: **Table S3.** Differentiated expressed proteins identified by proteomics analysis. (XLSX 271 kb)

Additional file 20: **Table S4.** Primers used in this study. (XLSX 13 kb)

## Data Availability

The data sets supporting the results of this article are included within the article and its additional files. The RNA-seq raw data used in this manuscript can be found in database of NCBI (https://www.ncbi.nlm.nih.gov/) under the following accession number: PRJNA557518. The mass spectrometry proteomics data have been deposited to the ProteomeXchange Consortium (http://proteomecentral.proteomexchange.org) via the iProX partner repository with the dataset identifier PXD‍015063.

## Abbreviations

CaMV: Cauliflower Mosaic Virus
CRISPR: Clustered Regularly Interspaced Short Palindromic Repeats
DSC: Distal Stem Cell
EMS: Ethyl methanesulfonate
FDR: False Discovery Rate
FKPM: fragments per kilobase per million reads
GO: Gene Ontology
GUS: β-Glucuronidase
IAA: Indole-3-acetic acid
InDel: Insertion and Deletion
KEGG: Kyoto Encyclopedia of Genes and Genomes
MS: Mass Spectrometry
NO: Nitric Oxide
PCD: Programmed Cell Death
PHBs: Prohibitins
QC: Quiescent Center Cell
QTL: Quantitative Trait Loci
SA: Salicylic Acid
SPFH: Stomatin, prohibitin, flotillin, HflK/C
SSR: Simple Sequence Repeats
TEM: Transmission electron microscopy
