# Supplementary figures and images for: NAL8 encodes a prohibitin that contributes to leaf and spikelet development by regulating mitochondria and chloroplasts stability in rice
Source: BMC Plant Biol. 2019 Sep 11;19:395. doi: 10.1186/s12870-019-2007-4 (PMC6737680; doi:10.1186/s12870-019-2007-4)

A

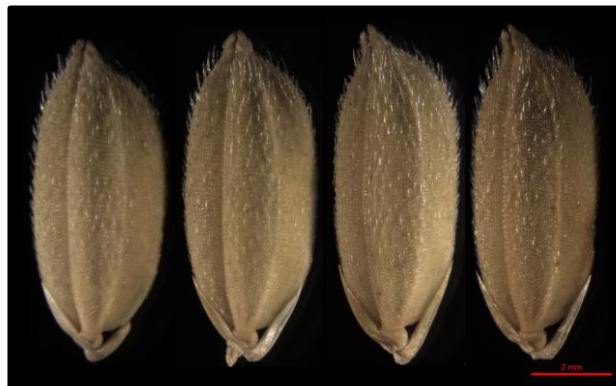

TQ

*nal8*

B

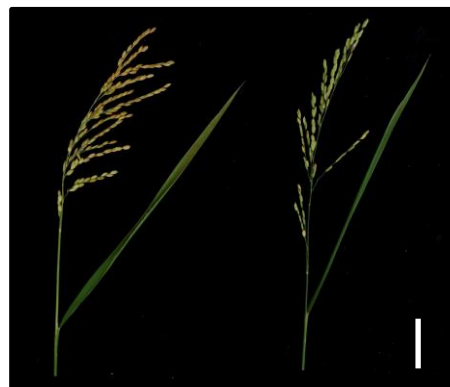

TQ

*nal8*

C

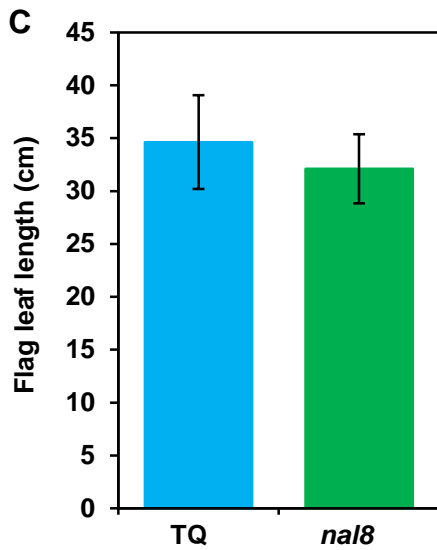

D

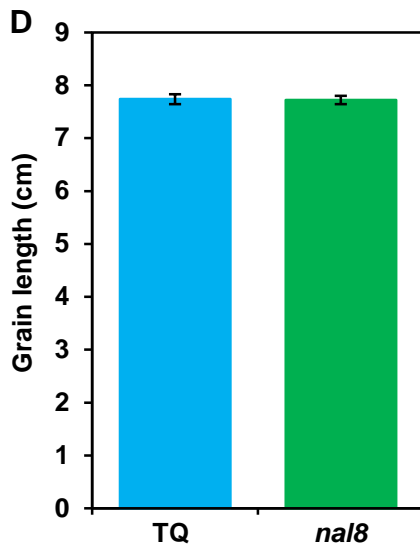

E

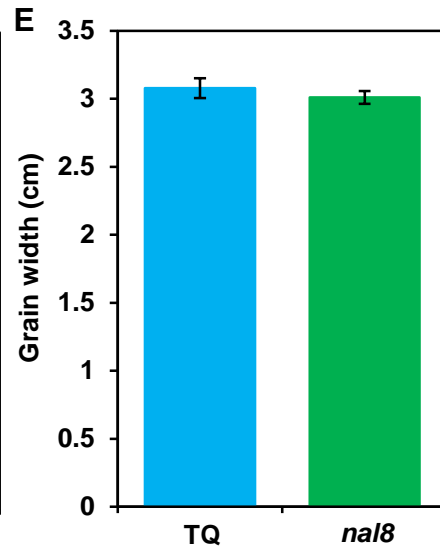

Supplement: Supplementary file 1 — Figure S1. Morphology and statistical analysis of mature grains and leaf length in TQ and the nal8 mutant. (A) Phenotypes of mature grains of TQ and the nal8 mutant. Scale bar = 2 mm. (B) Comparison of the main spikelets from TQ and the nal8 mutant. Scale bar = 2 cm. (C-E) Statistical analyses of average flag leaf length (n = 20 plants) (C), average grain length (n = 20 plants) (D) and average grain width (n = 20 plants) (E) between TQ and the nal8 mutant. Values are given as the mean ± SD. *P < 0.05; **P < 0.01 compared with the TQ control using Student’s t-test (C-E). (PDF 57 kb) [file 12870_2019_2007_MOESM1_ESM.pdf]

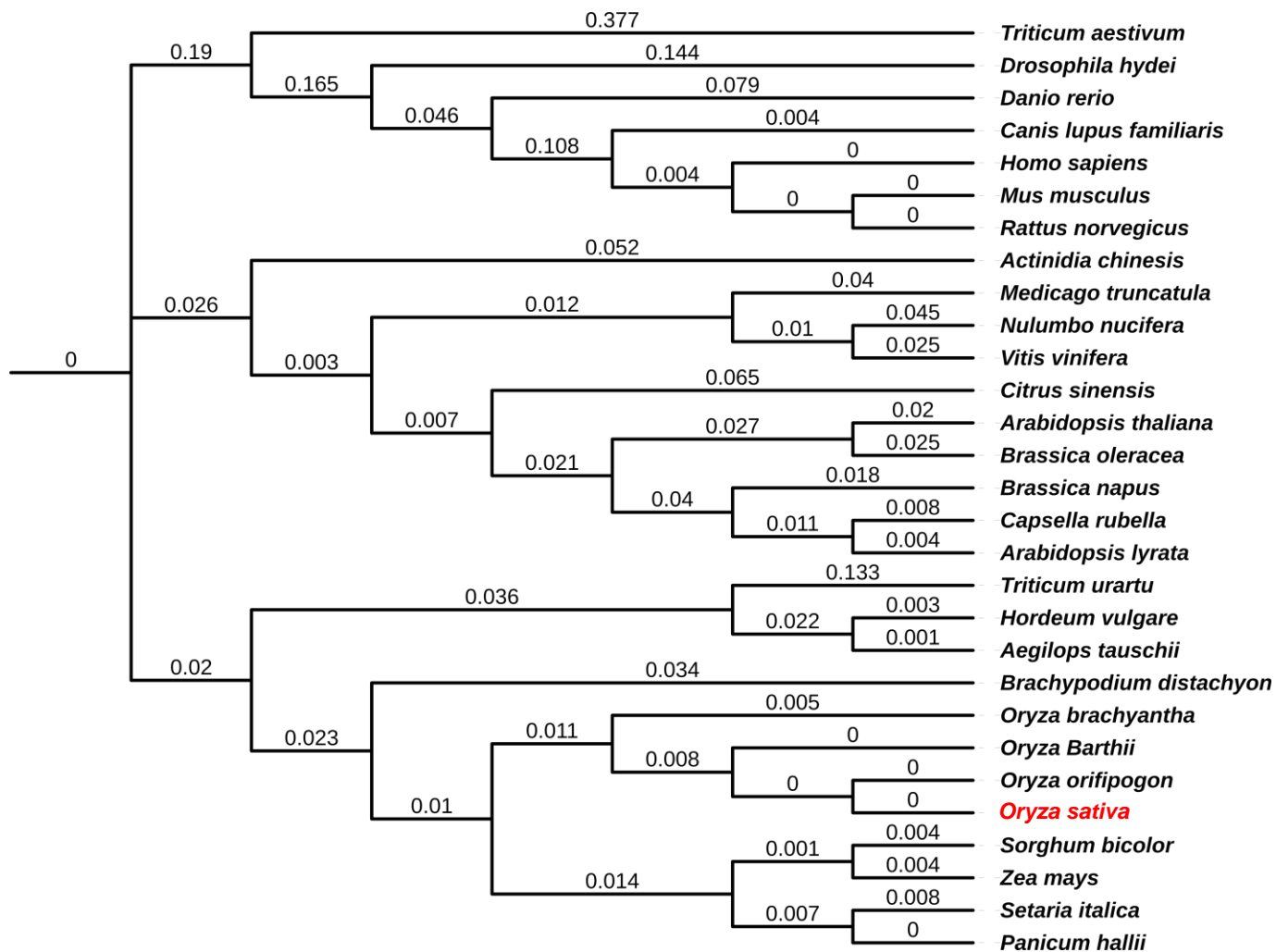

Supplement: Supplementary file 3 — Figure S3. Phylogenetic analysis of rice NAL8 and related proteins from plants, animals, and one species of insect. The species names are shown at the ends of the branches. The phylogenetic tree was constructed using the Neighbor-Joining tree method as implemented in MEGA7.0 and embellished with iTOL (http://itol.embl.de/). The numbers shown on each branch indicate protein substitution rate. The NAL8 homologous proteins are highly consistent with the species evolution relationship. (PDF 117 kb) [file 12870_2019_2007_MOESM3_ESM.pdf]

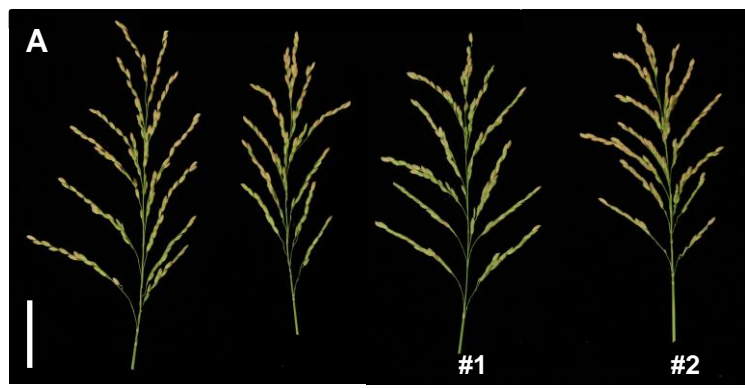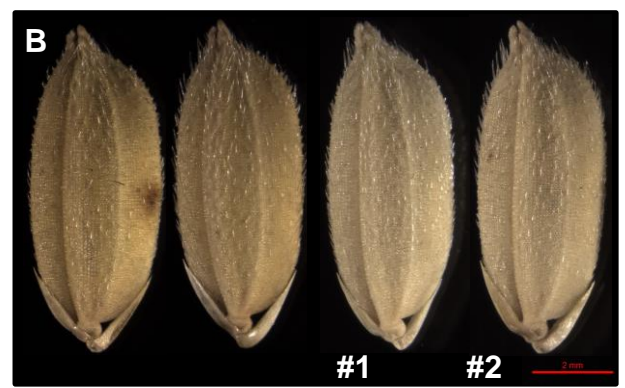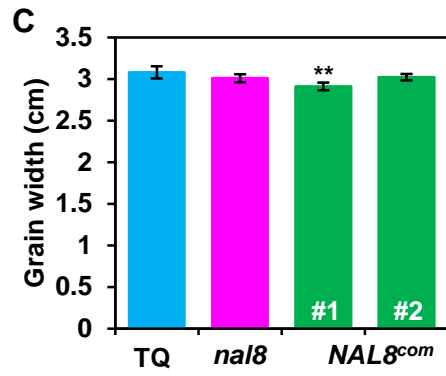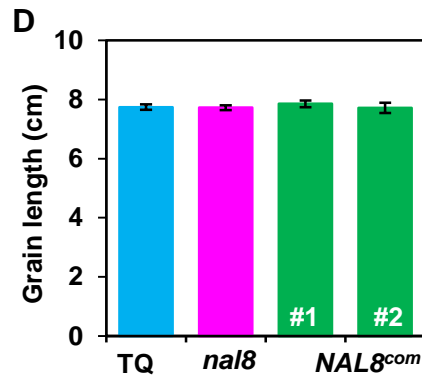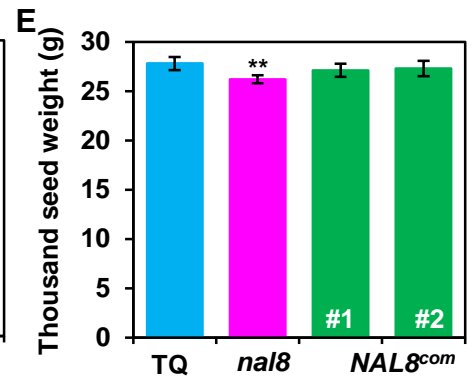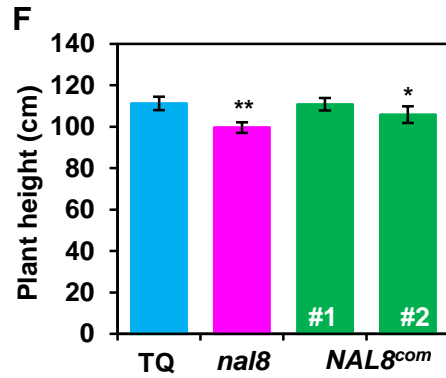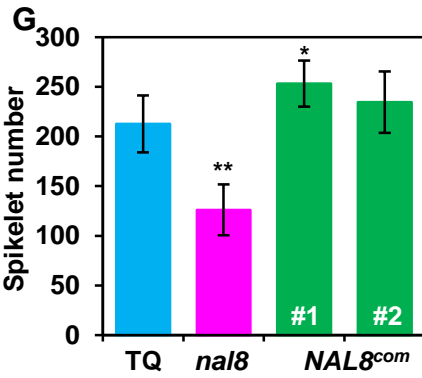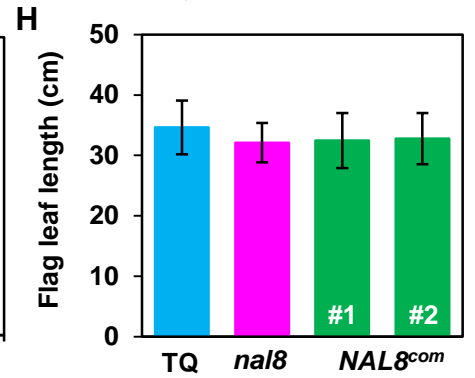

Supplement: Supplementary file 4 — Figure S4. Comparisons and statistical analysis of TQ, the nal8 mutant, and NAL8 complementation transgenic rice lines. (A) Spikelet phenotypes among TQ, nal8 and the transgenic complementation lines NAL8com#1 and #2. Scale bar = 5 cm. (B) Mature grains of TQ, nal8 and the complementation lines NAL8com#1 and #2. Scale bar = 2 mm. (C-H) Statistical analyses of average grain width (n = 20 plants) (C), average grain length (n = 20 plants) (D), average thousand seed weight (n = 20 plants) (E), average plant height (n = 20 plants) (F), average spikelet number (n = 20 plants) (G) and average flag leaf length (n = 20 plants) (H) in TQ, nal8 and the transgenic complementation lines NAL8com#1 and #2. Values in C-H are given as the mean ± SD. *P < 0.05; **P < 0.01 compared with the TQ control using Student’s t-test. (PDF 80 kb) [file 12870_2019_2007_MOESM4_ESM.pdf]

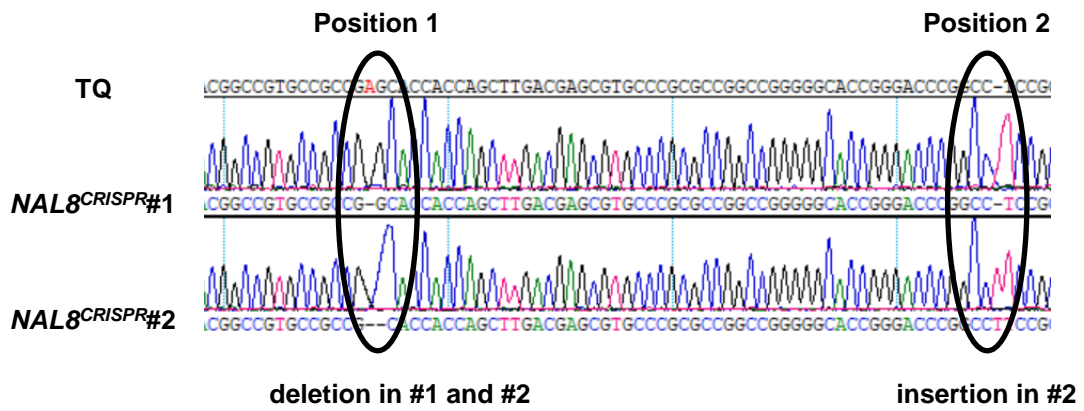

Supplement: Supplementary file 5 — Figure S5. Genotyping of the NAL8CRISPR transgenic knockout lines. Alignment of the DNA sequences of the NAL8 gene region from the wild-type TQ and the NAL8CRISPR #1 and #2 lines. Black ellipses show the positions of a 1 bp deletion in the NAL8CRISPR #1 and a 2 bp deletion in NAL8CRISPR # 2 sequences (left), and 1 bp insertion in NAL8CRISPR # 2 (right). The mutations cause translational frame shifts which result in missense mutations in the NAL8 gene in both transgenic knockout lines. (PDF 49 kb) [file 12870_2019_2007_MOESM5_ESM.pdf]

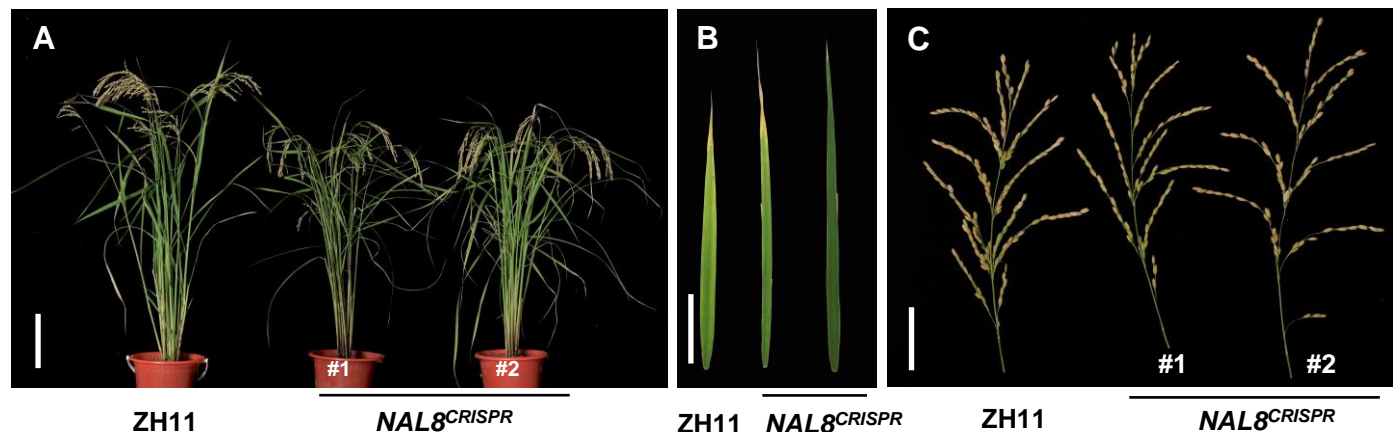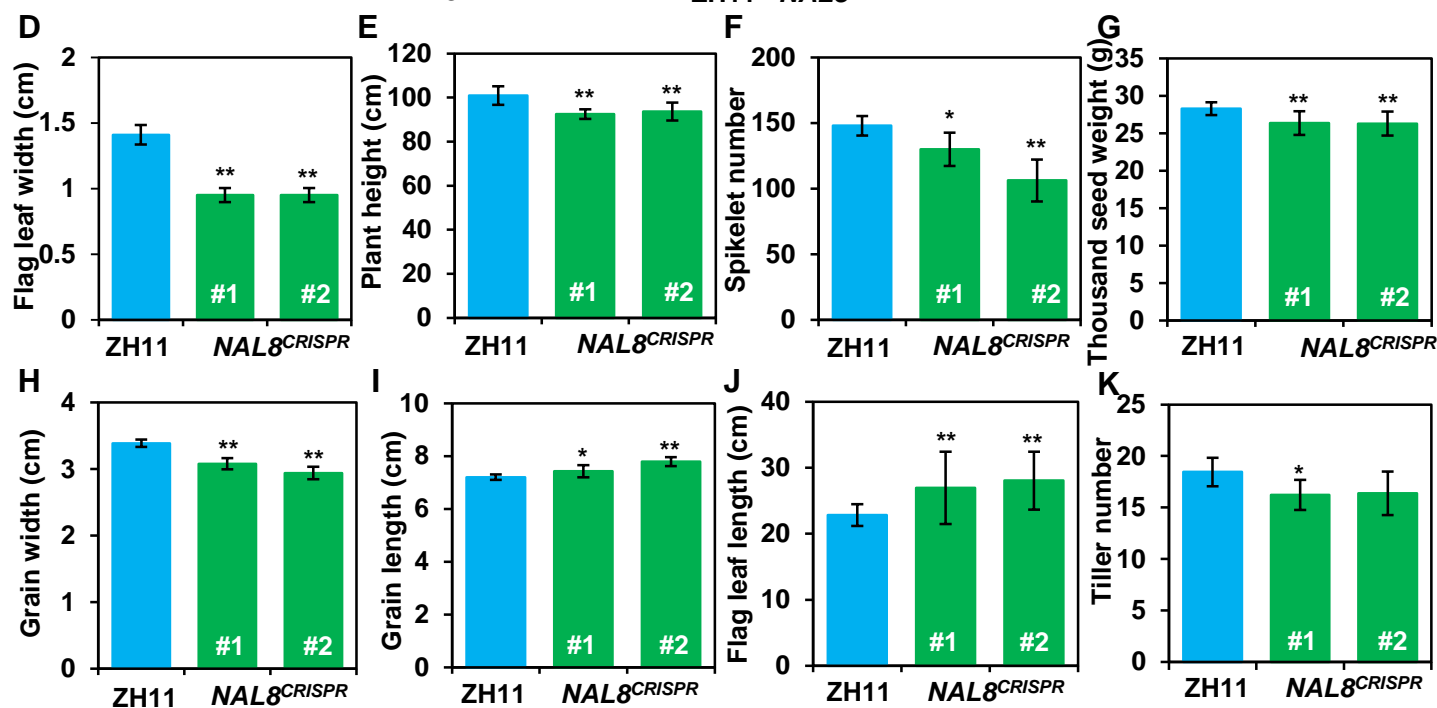

Supplement: Supplementary file 6 — Figure S6. Narrow leaf width and reduced spikelet number in the NAL8 transgenic knockout lines are similar to those in the nal8 mutant. (A) Plant architecture of the wild-type ZH11 and the NAL8 knockout lines NAL8CRISPR #1 and #2 at the reproductive stage. Scale bar = 15 cm. (B) Flag leaves of ZH11 and NAL8CRISPR #1 and #2. Scale bar = 5 cm. (C) Mature panicles of ZH11 and NAL8CRISPR #1 and #2. Scale bar = 10 cm. (D-K) Statistical comparisons of the average flag leaf width (n = 20 plants) (D), average plant height (n = 20 plants) (E), average spikelet number (n = 20 plants) (F), average thousand seed weight (n = 20 plants) (G), average grain width (n = 20 plants) (H), average grain length (n = 20 plants) (I), average flag leaf length (n = 20 plants) (J) and average tiller number (n = 20 plants) (K) in ZH11 and the NAL8CRISPR #1 and #2 knockout lines. Values in D-K are given as the mean ± SD. *P < 0.05; **P < 0.01 compared with the ZH11 control using Student’s t-test. (PDF 79 kb) [file 12870_2019_2007_MOESM6_ESM.pdf]

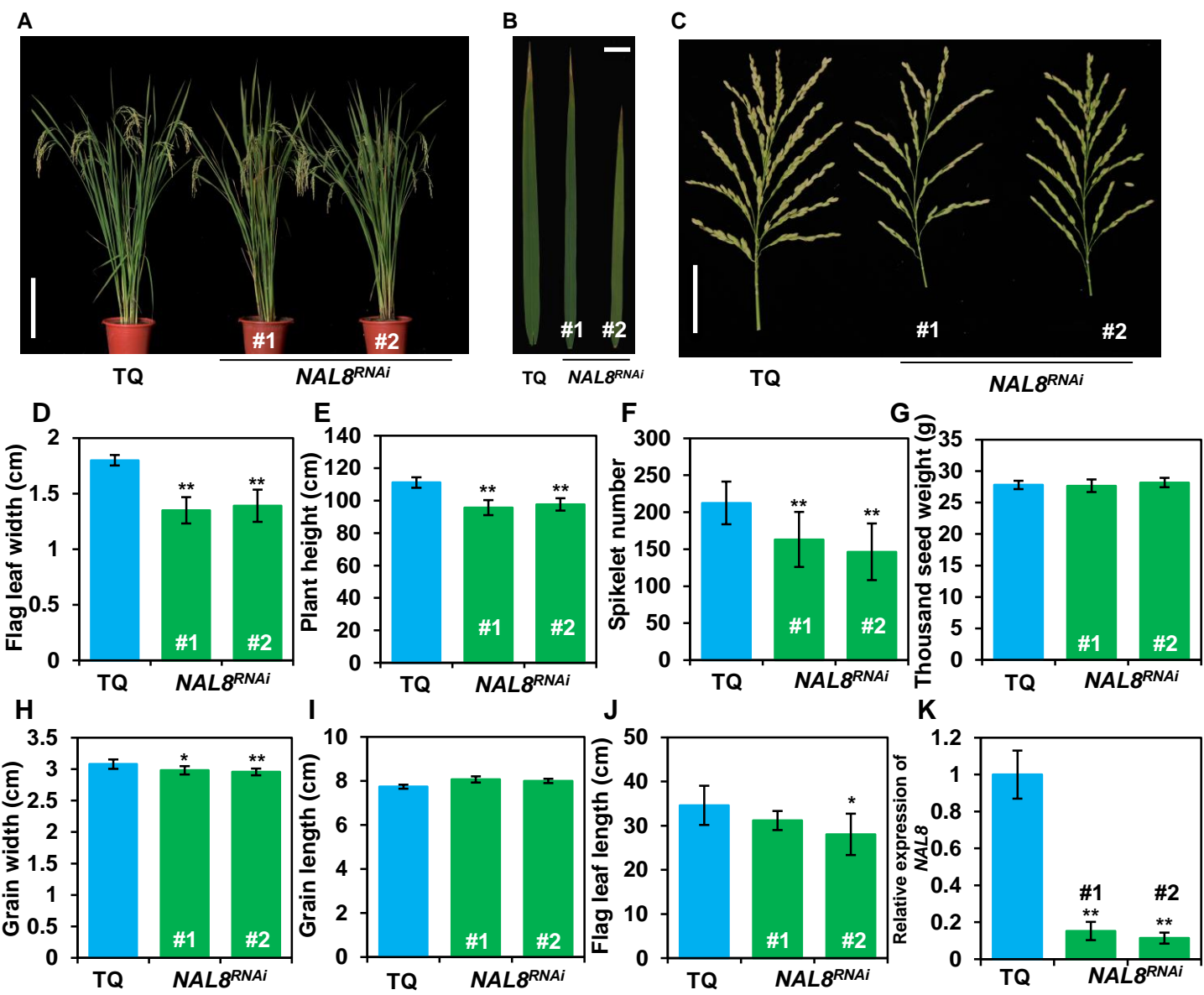

Supplement: Supplementary file 7 — Figure S7. Transgenic NAL8 gene silenced lines show similar leaf width and spikelet defects to the rice nal8 mutant. (A) Plant architecture of the wild-type TQ and the transgenic NAL8 RNAi silenced lines NAL8RNAi #1 and #2 at the reproductive stage. Scale bar = 15 cm. (B) Flag leaves of TQ and NAL8RNAi #1 and #2. Scale bar = 5 cm. (C) Mature panicles of ZH11 and NAL8RNAi #1 and #2. Scale bar = 5 cm. (D-K) Statistical comparisons of average flag leaf width (n = 20 plants) (D), average plant height (n = 20 plants) (E), average spikelet number (n = 20 plants) (F), average thousand seed weight (n = 20 plants) (G), average grain width (n = 20 plants) (H), average grain length (n = 20 plants) (I), average flag leaf length (n = 20 plants) (J) and the relative expression of NAL8 (n = 3 pooled tissues, three plants per pool) (K) in ZH11 and NAL8RNAi #1 and #2. Values in D-K are given as the mean ± SD. *P < 0.05; **P < 0.01 compared with the TQ control using Student’s t-test. The UBQ5 gene was used as an internal reference to normalize the gene expression data. (PDF 81 kb) [file 12870_2019_2007_MOESM7_ESM.pdf]

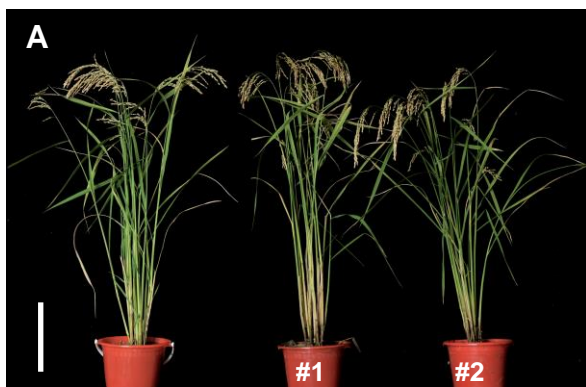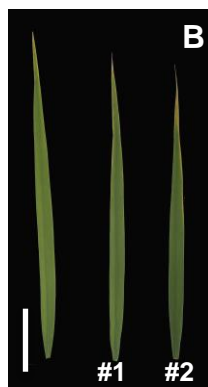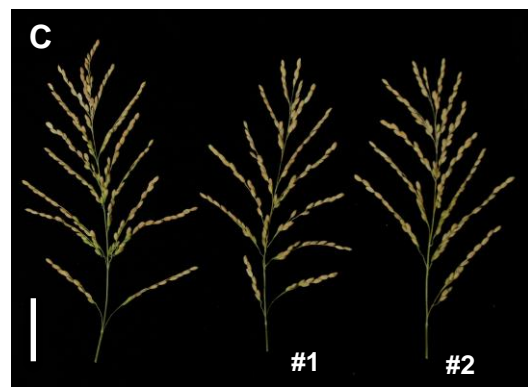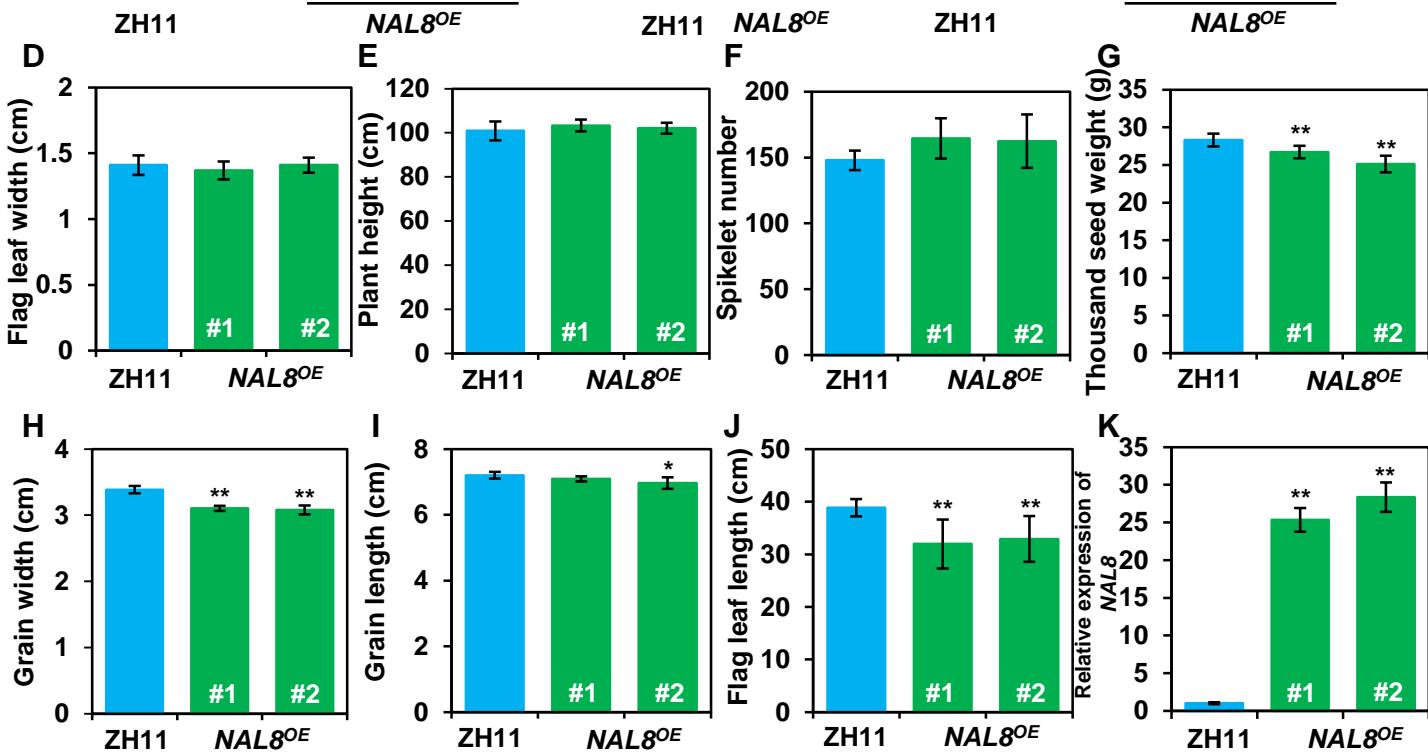

Supplement: Supplementary file 8 — Figure S8. Transgenic rice plants overexpressing NAL8 show no obvious developmental effects. (A) Plant architecture of the wild-type ZH11 and transgenic NAL8-overexpressing lines NAL8OE #1 and #2 at the reproductive stage. Scale bar = 15 cm. (B) Flag leaves of ZH11 and NAL8OE #1 and #2. Scale bar = 5 cm. (C) Mature panicles of ZH11 and NAL8OE #1 and #2. Scale bar = 5 cm. (D-K) Statistical comparisons of average flag leaf width (n = 20 plants) (D), average plant height (n = 20 plants) (E), average spikelet number (n = 20 plants) (F), average thousand seed weight (n = 20 plants) (G), average grain width (n = 20 plants) (H), average grain length (n = 20 plants) (I), average flag leaf length (n = 20 plants) (J) and the relative expression of NAL8 (n = 3 pooled tissues, three plants per pool) (K) in ZH11 and NAL8OE #1 and #2. Values in (D-K) are given as the mean ± SD. *P < 0.05; **P < 0.01 compared with the ZH11 control using Student’s t-test. The UBQ5 gene was used as an internal reference to normalize the gene expression data. (PDF 79 kb) [file 12870_2019_2007_MOESM8_ESM.pdf]

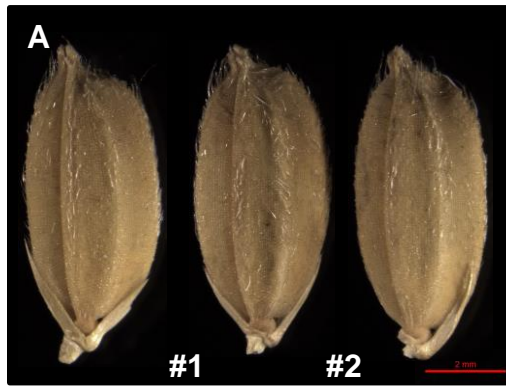

ZH11

*NAL8*<sup>CRISPR</sup>

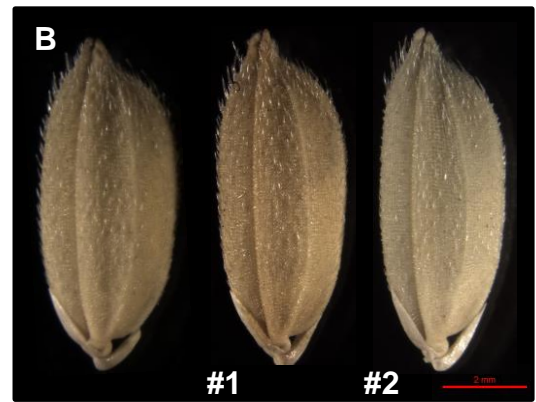

TQ

*NAL8*<sup>RNAi</sup>

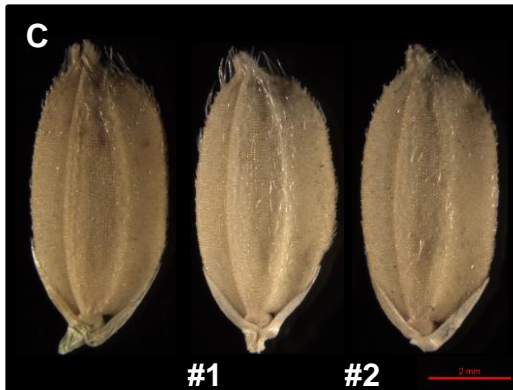

ZH11

*NAL8*<sup>OE</sup>

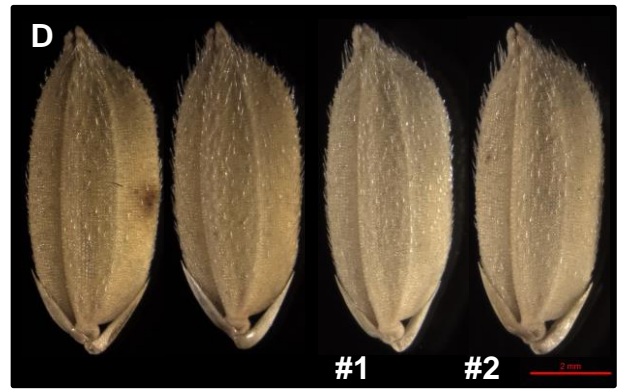

TQ

*nal8*

*NAL8*<sup>com</sup>

Supplement: Supplementary file 9 — Figure S9. Grain phenotypes of the NAL8 transgenic lines. (A) Mature rice grains of ZH11 and the transgenic NAL8OE overexpression lines. Scale bar = 2 mm. (B) Mature rice grains of TQ and the transgenic NAL8RNAi gene silencing lines. Scale bar = 2 mm. (C) Mature rice grains of ZH11 and the transgenic NAL8CRISPR transgenic lines. Scale bar = 2 mm. (D) Mature rice grains of TQ, the nal8 mutant and the transgenic two NAL8com complementation lines in the nal8 genetic background. Scale bar = 2 mm. (PDF 118 kb) [file 12870_2019_2007_MOESM9_ESM.pdf]

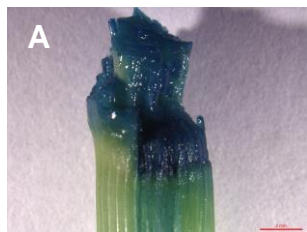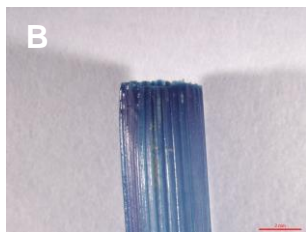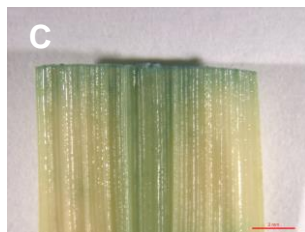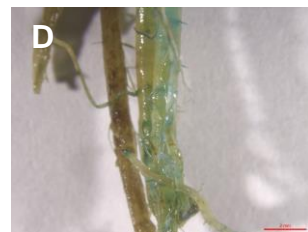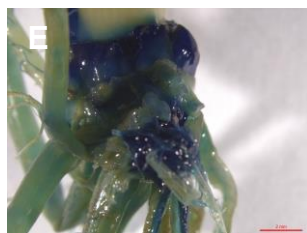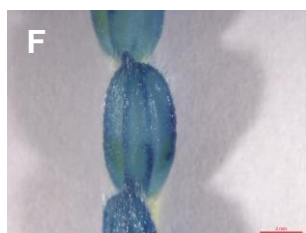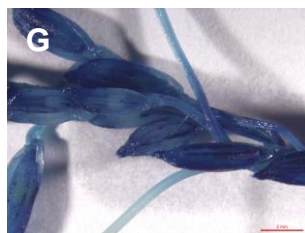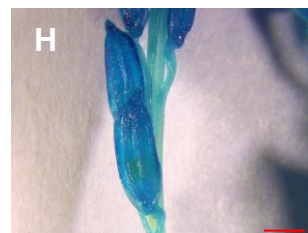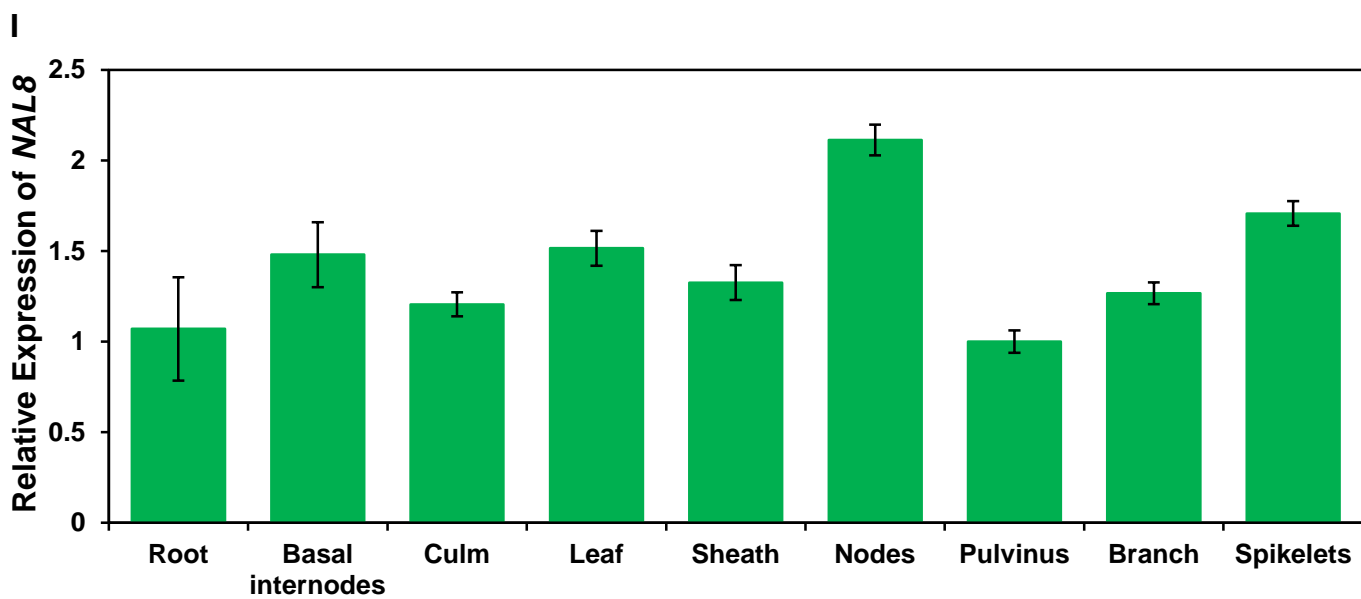

Supplement: Supplementary file 10 — Figure S10. Expression profile of NAL8 in rice tissues and organs. (A-H) Histochemical analysis of enzyme activity of the NAL8:GUS fusion protein in the node (A), culm (B), leaf (C), root (D), basal internode (E), spikelet hull (F), young panicle (G), and in transgenic plants expressing the GUS gene under control of the NAL8 promoter (H). Scale bar = 2 mm. (I) Relative expression of the NAL8 gene in different rice tissues. The UBQ5 gene was used as the internal reference to normalize gene expression data. The standard deviations were calculated from three biological replicates. (PDF 83 kb) [file 12870_2019_2007_MOESM10_ESM.pdf]

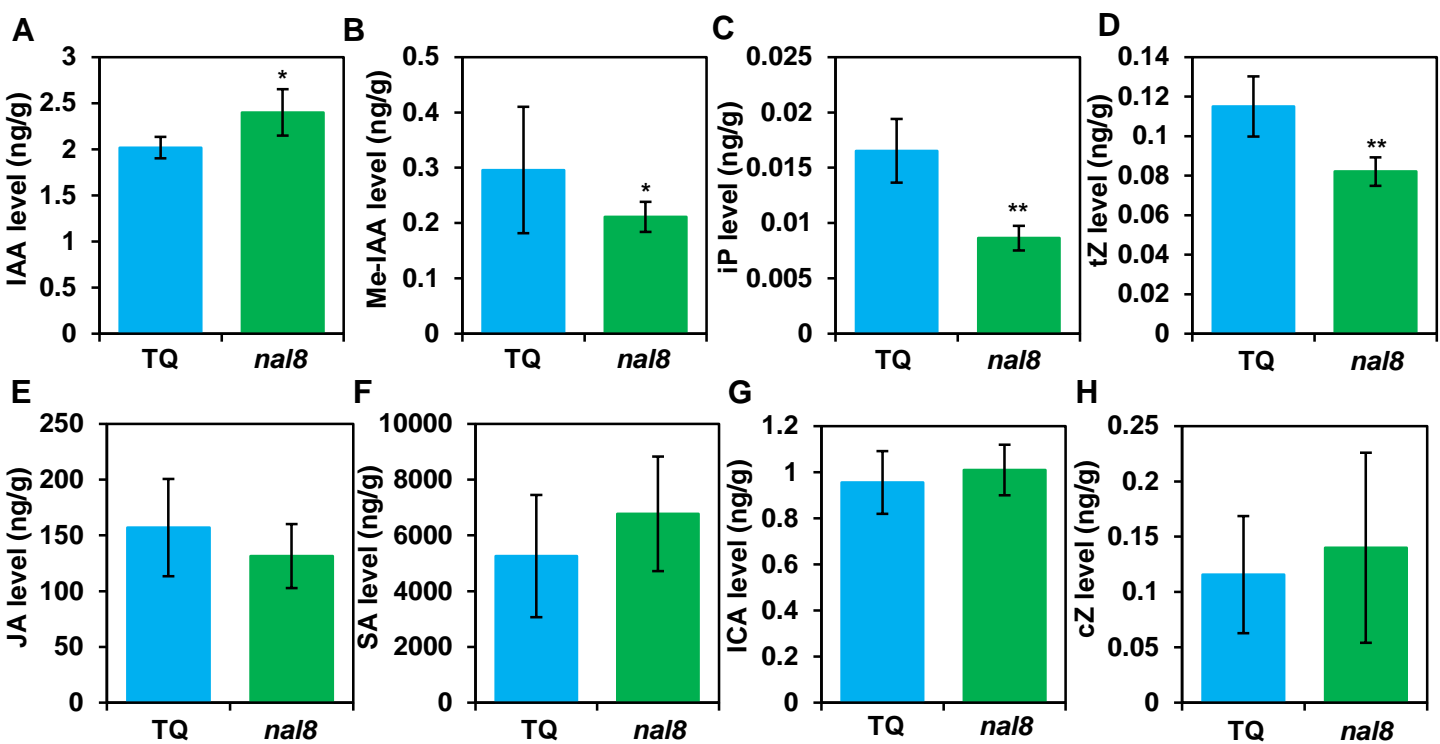

Supplement: Supplementary file 11 — Figure S11. Endogenous levels of multiple plant hormones in TQ and the nal8 mutant. (A-H) Endogenous levels of IAA (A), Me-IAA (B), iP (C), tZ (D), JA (E), SA (F), ICA (G) and cZ (H) in TQ and the nal8 mutant. Standard deviations were calculated from three biological replicates. (PDF 12 kb) [file 12870_2019_2007_MOESM11_ESM.pdf]

A

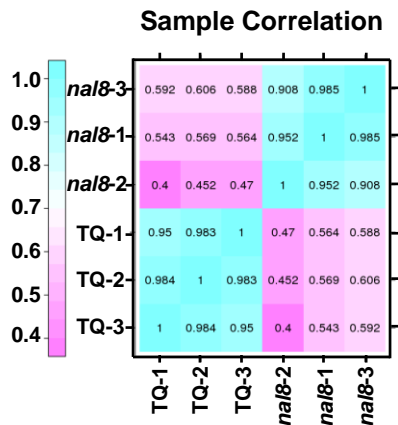

B

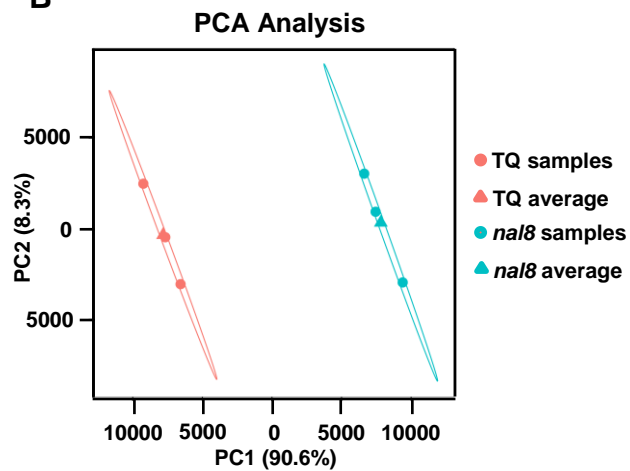

C

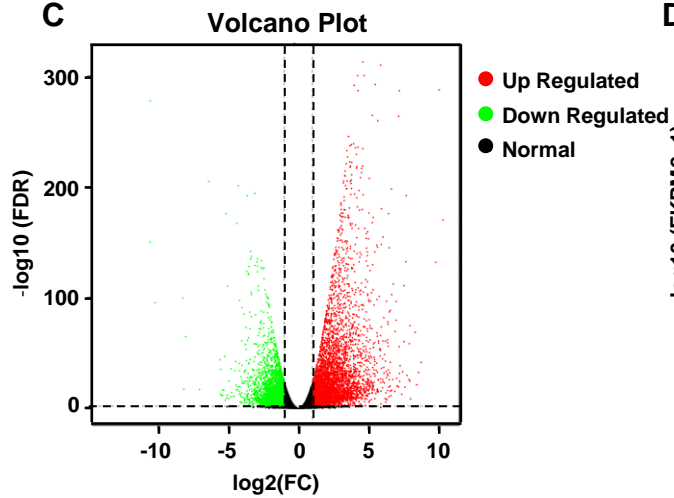

D

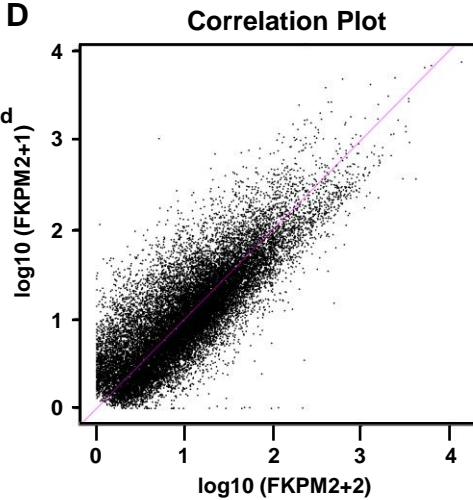

Supplement: Supplementary file 12 — Figure S12. Comparative RNA-seq analysis between TQ and the nal8 mutant. (A) Sample correlations among the individual TQ and nal8 replicates and between TQ and nal8. Each sample consisted of three biological replicates. The numbers indicate the pearson’s correlation coefficients for each pair-wise comparison. (B) A PCA map indicates that the TQ and nal8 samples are widely separated in the RNA-seq analysis. (C) A volcano plot shows that more genes are up-regulated in the nal8 mutant than are down-regulated compared with TQ. The X-axes shows the logarithmic values of the relative differences in expression. The Y-axes values are the negative logarithms of the significance. Each dot indicates a gene. Red dots and green dots are significantly up-regulated and down-regulated genes, respectively. Black dots are genes in which the expression changes are insignificant. The expression values for the different RNAs were adjusted by FDR < 0.01, and the Fold Change is > 2. (D) Correlation plot of all TQ and nal8 samples which were used in the RNA-seq analysis. The X- and Y-axes are the logarithmic values of FKPM (fragments per kilobase per million reads). Each dot indicates a gene. The purple line is the coefficient parameter which represents the Pearson’s coefficient of significance, above which the genes are up-regulated, and below which the genes are down-regulated. (PDF 314 kb) [file 12870_2019_2007_MOESM12_ESM.pdf]

A

## KEGG Classification

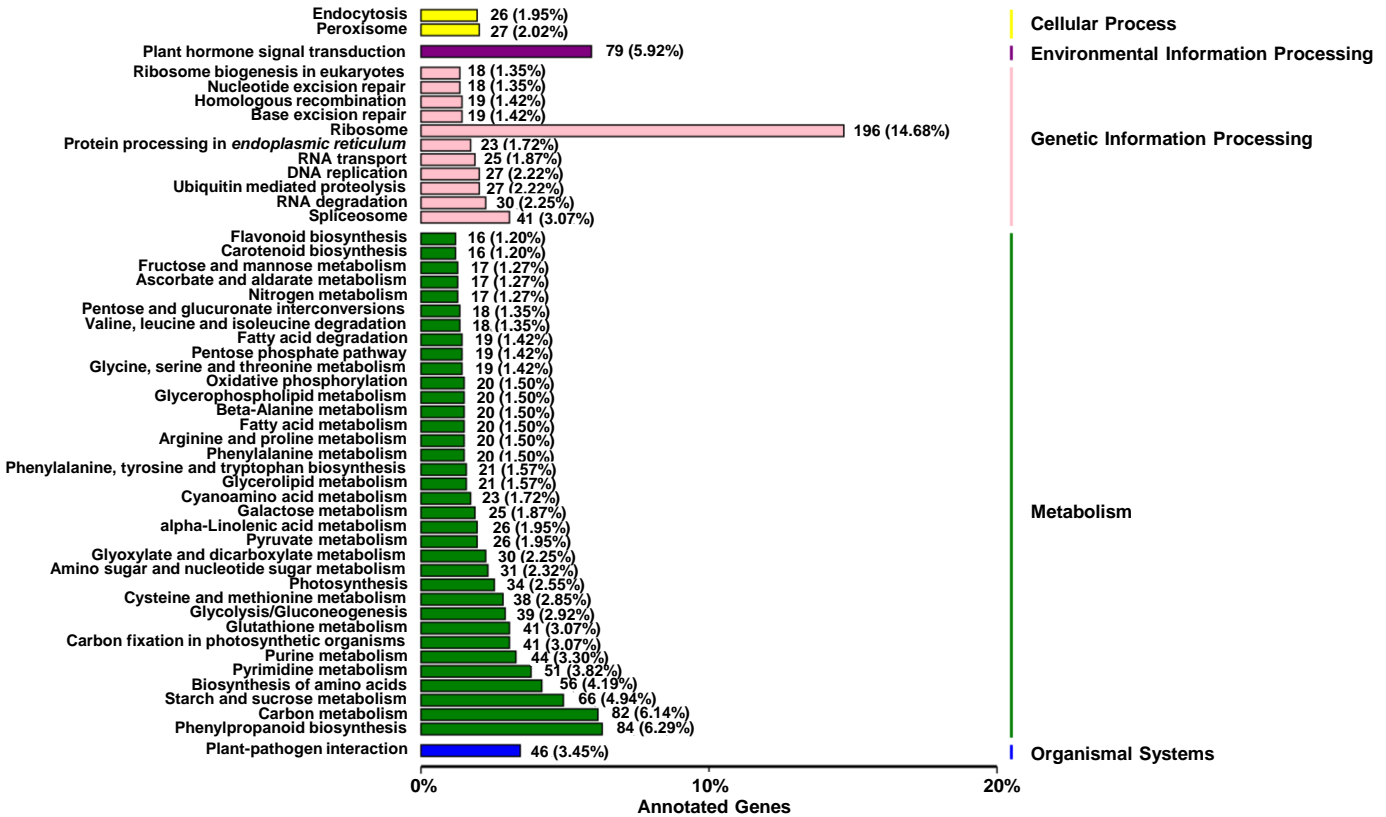

B

## GO Enrichment

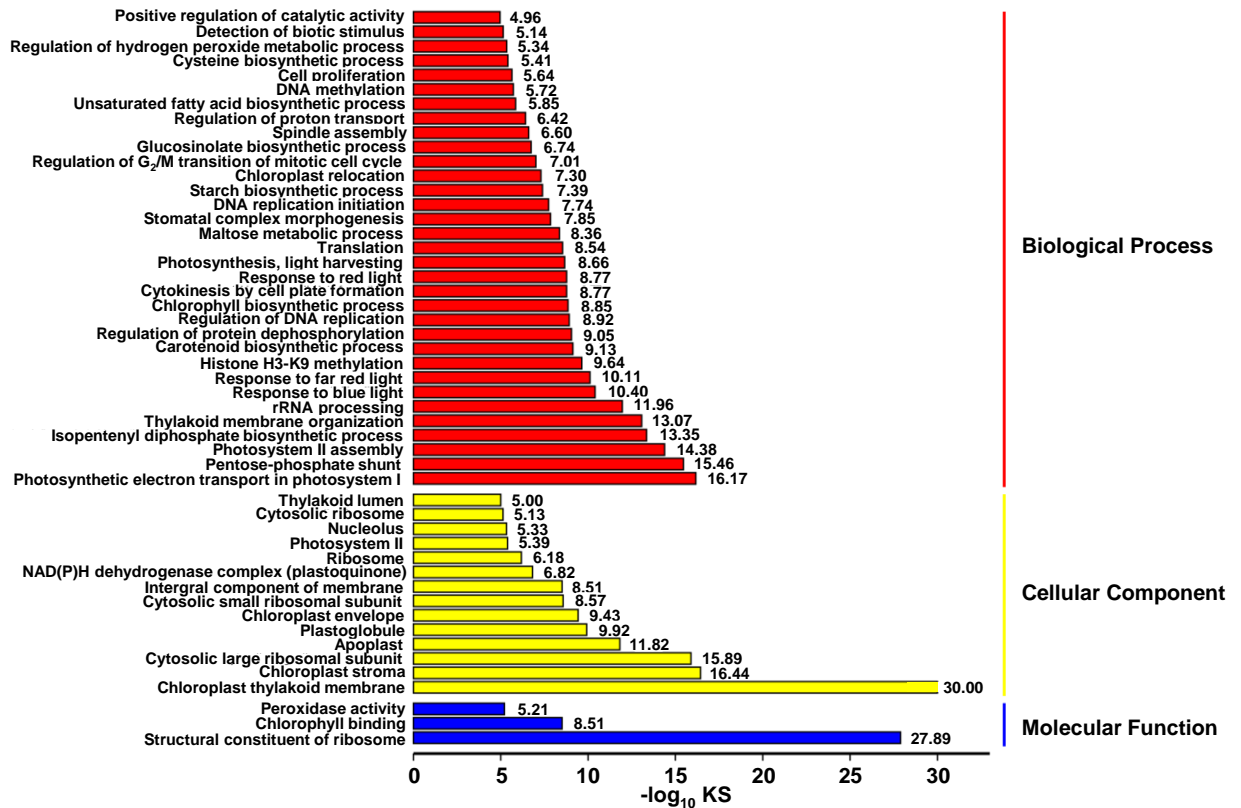

Supplement: Supplementary file 13 — Figure S13. KEGG pathway analysis and GO analysis for genes that are differentially expressed between TQ and the nal8 mutant. (A) All differentially-expressed genes (DEGs) that gave a match in the KEGG (Kyoto Encyclopedia of Genes and Genomes) classification. The numbers and percentages of annotated genes are shown to the right, and the various colors indicate the main KEGG pathway classifications. The x-axes indicates the percentage of annotated genes out of all DEGs. (B) GO (gene ontology) enrichment of DEGs between TQ and nal8. The individual GO terms in the three main GO categories are shown on the y-axes, and the x-axes shows the log10 of the significance of the corresponding GO terms. (PDF 211 kb) [file 12870_2019_2007_MOESM13_ESM.pdf]

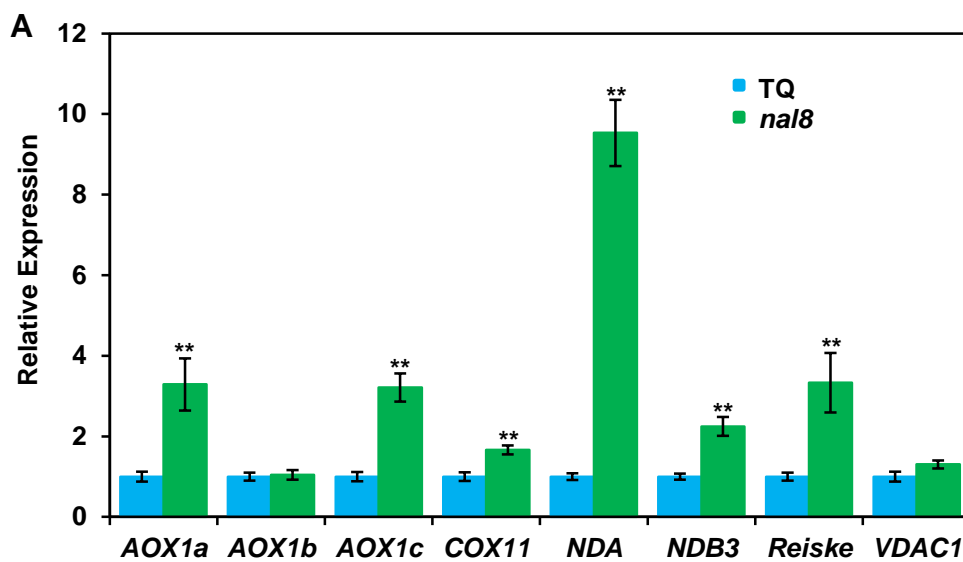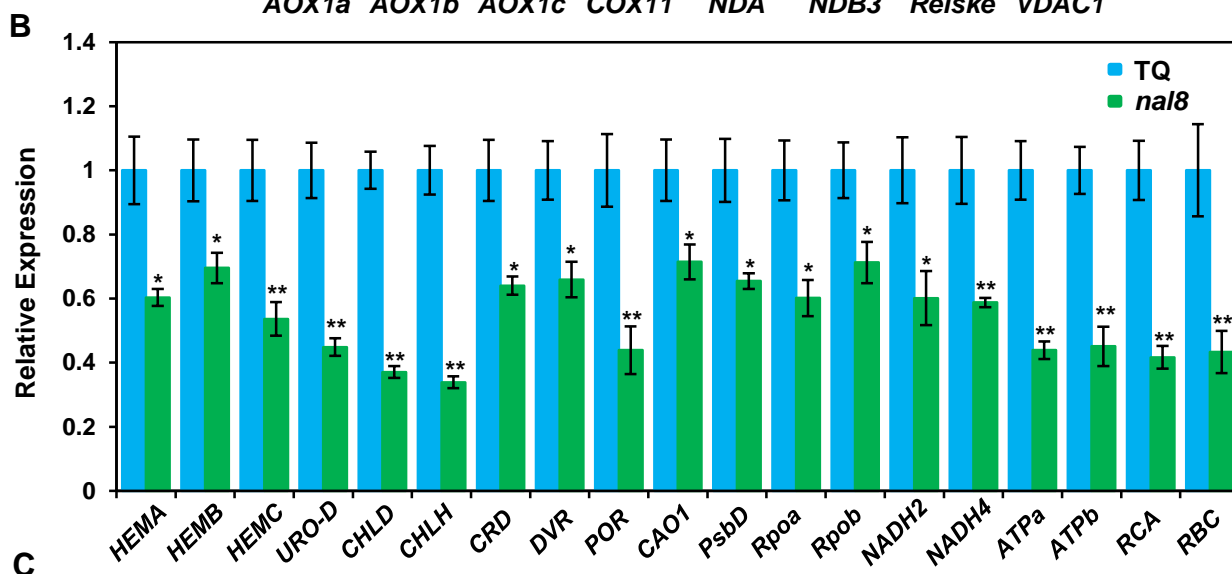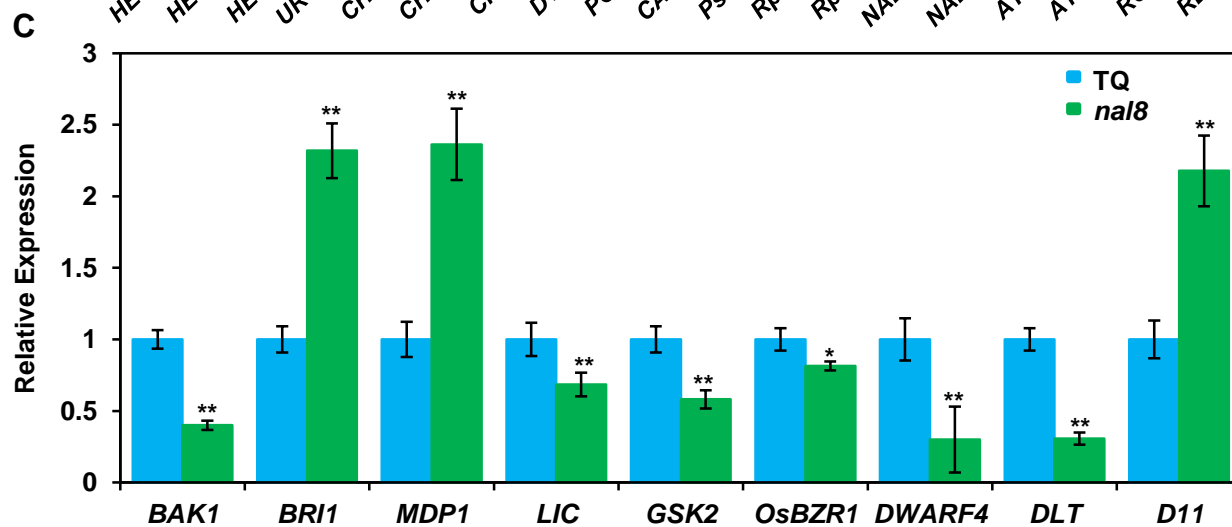

Supplement: Supplementary file 14 — Figure S14. qRT-PCR analysis of gene expression between TQ and the nal8 mutant. (A-C) Relative expression levels of mitochondrial genes (A), chloroplast-related genes (B) and brassinosteroid-related genes (C) in TQ and nal8 as determined by qRT-PCR (n = 3 pooled tissues). The UBQ5 gene was used as an internal reference to normalize the gene expression data. Values represent the mean ± SD. **P < 0.01 compared with the wild-type using Student’s t-test. (PDF 46 kb) [file 12870_2019_2007_MOESM14_ESM.pdf]

**A**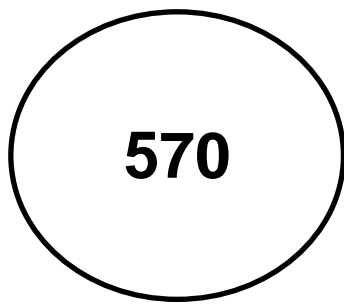

upregulated

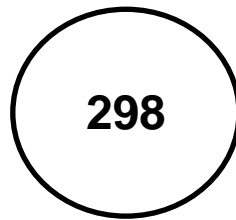

downregulated

TQ vs. *nal8***B**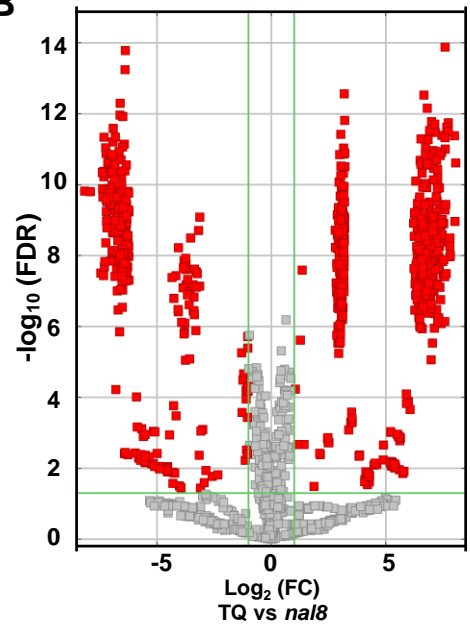

Supplement: Supplementary file 15 — Figure S15. Comparative proteomics analysis of TQ and the nal8 mutant. (A) Venn diagram showing the number of different proteins that are up- and down-regulated in TQ compared with nal8. The expression values for the different proteins were adjusted by FDR (false discovery rate). Three biological repeats of both TQ and nal8 were used to perform the proteomics analysis. (B) Volcano plot showing the distribution of differential protein expression. The x-axes shows the Log2 fold-change of the differentially expressed proteins, and the y-axes shows the-log10 of the p-values of the differences between TQ and nal8. The expression values for the different proteins were adjusted by FDR < 0.01, and the Fold Change is > 2. (PDF 227 kb) [file 12870_2019_2007_MOESM15_ESM.pdf]

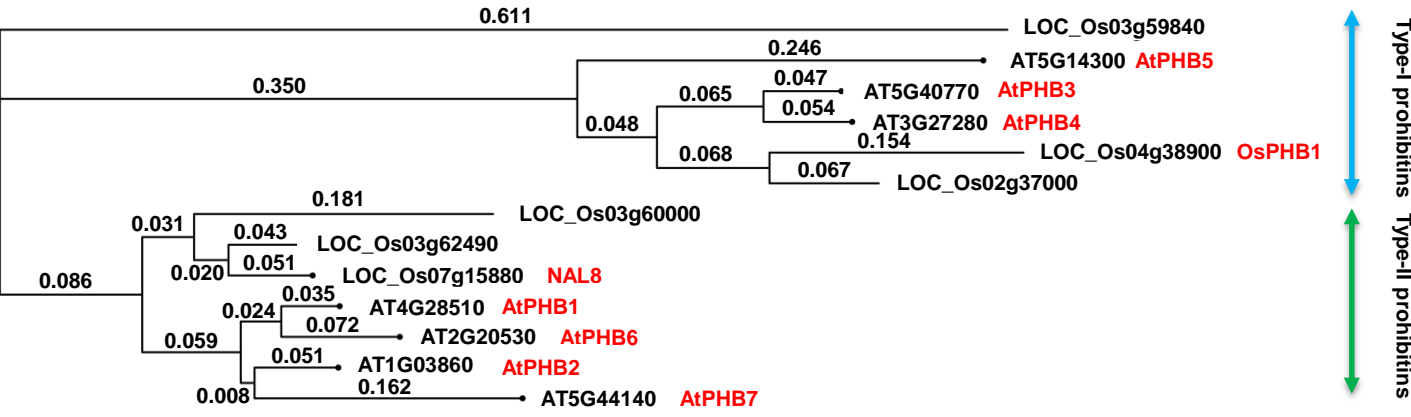

Supplement: Supplementary file 16 — Figure S16. Phylogenetic analysis of the PHB proteins from rice and Arabidopsis. Phylogenetic analysis of NAL8 and homologous protein sequences from rice (Os) and Arabidopsis (At). The gene and/or locus names are shown at the ends of the branches on the phylogenetic tree. The tree was constructed using the Neighbor-Joining method as implemented in MEGA7.0 and embellished with iTOL (http://itol.embl.de/). The numbers shown on each branch indicate protein substitution rate. NAL8 belongs to the type-II class of prohibitins. (PDF 40 kb) [file 12870_2019_2007_MOESM16_ESM.pdf]
