# Supplementary material for: NAL8 encodes a prohibitin that contributes to leaf and spikelet development by regulating mitochondria and chloroplasts stability in rice
Source: BMC Plant Biol. 2019 Sep 11;19:395. doi: 10.1186/s12870-019-2007-4 (PMC6737680; doi:10.1186/s12870-019-2007-4)
Supplement: Supplementary file 2 — Figure S2. Amino acid sequence alignment of homologous proteins to rice NAL8. The conserved alanine residue at position 228 is enclosed in a red box to show the site of the mutation in the nal8 mutant. The asterisks indicate the highly conserved residues in the proteins from nine plants, three mammals and one insect species. (PDF 668 kb) [file 12870_2019_2007_MOESM2_ESM.pdf]

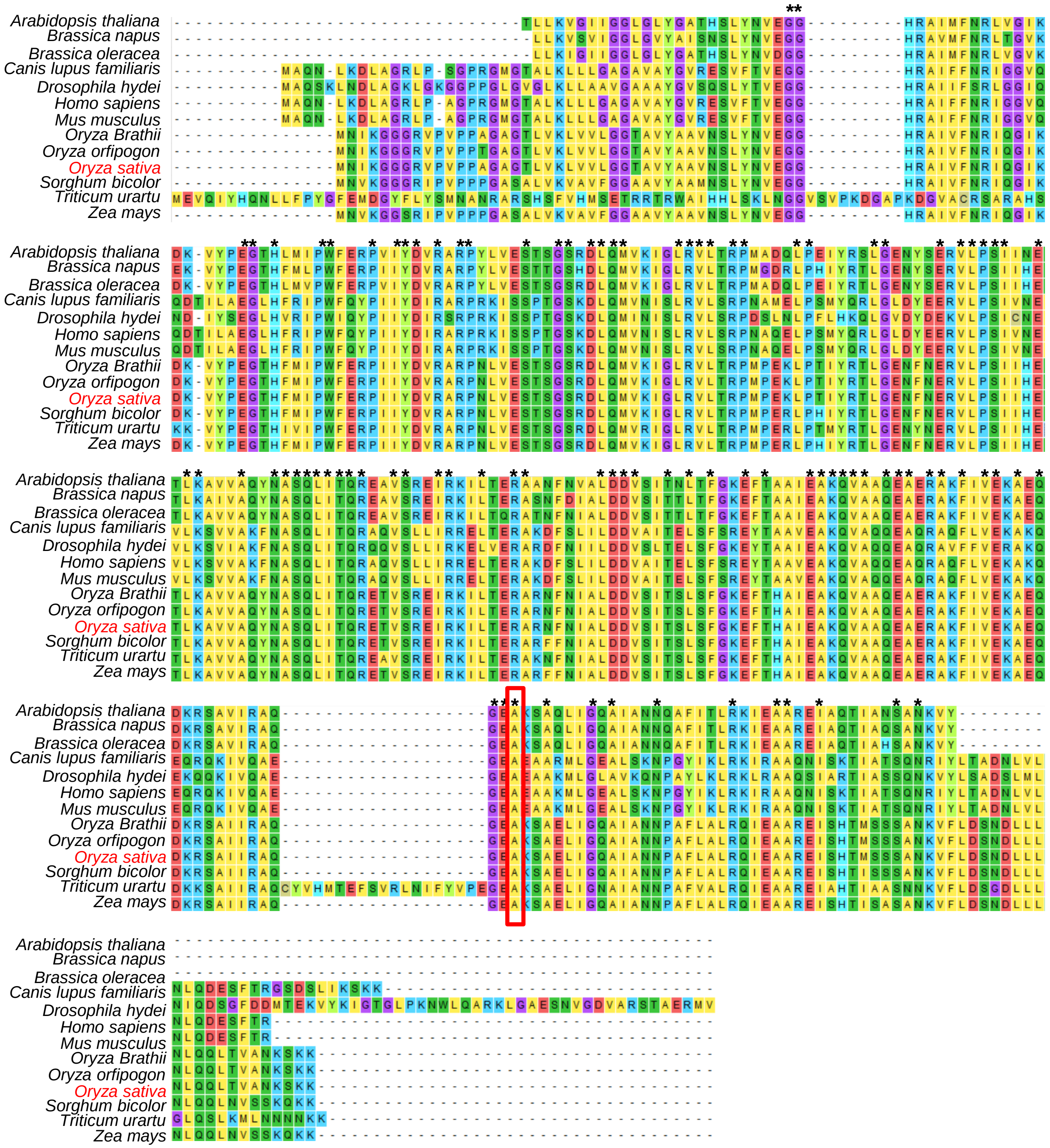
Arabidopsis thaliana
Brassica napus
Brassica oleracea
Canis lupus familiaris
Drosophila hydei
Homo sapiens
Mus musculus
Oryza Brathii
Oryza orfipogon
Oryza sativa
Sorghum bicolor
Triticum urartu
Zea mays
